# A Tutorial for Scanning Electrochemical Cell Microscopy (SECCM) Measurements: Step-by-Step Instructions, Visual Resources, and Guidance for First Experiments

**DOI:** 10.1021/acsmeasuresciau.4c00091

**Published:** 2025-03-27

**Authors:** Kamsy Lerae Anderson, Martin Andrew Edwards

**Affiliations:** Department of Chemistry and Biochemistry, University of Arkansas, Fayetteville, Arkansas 72701, United States

**Keywords:** electrochemical imaging, nanopipette, nanoelectrochemistry, scanned probe microscopy, nanodroplet, topography, scanning micropipet contact method (SMCM), single-entity electrochemistry, structure−activity relationships

## Abstract

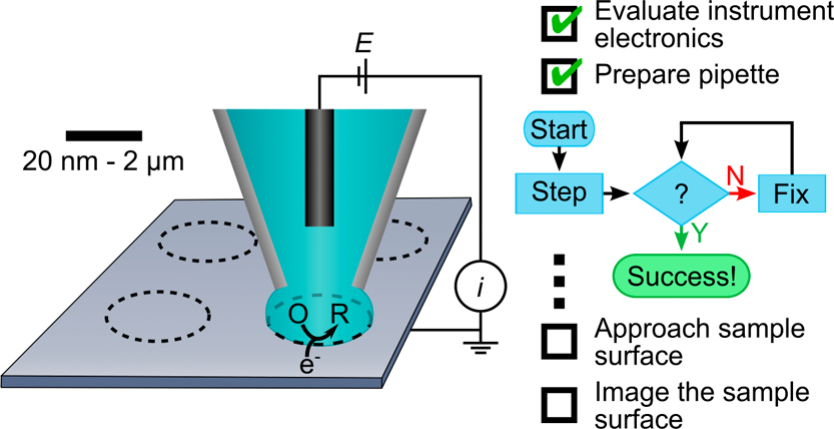

Scanning electrochemical cell microscopy (SECCM) produces nanoscale-resolution electrochemical maps of electrode surfaces using the meniscus at the tip of an electrolyte-filled nanopipette as a mobile electrochemical cell. While the use and range of applications of SECCM have grown rapidly since its introduction, the pathway to performing SECCM measurements can be daunting to those without direct access to expert users. This work fills this expertise gap by providing a step-by-step guide to performing one’s first SECCM experiments, including troubleshooting strategies, videos/images, suggested parameters and experimental systems, and representative data (of both successful experiments and common problems). No background in SECCM is assumed and fundamentals are clearly explained at each stage with a rationale for the experimental steps provided. This work provides an entry point for the uninitiated to understand and use this powerful nanoscale electrochemical characterization technique.

## Introduction

**Figure 1:**
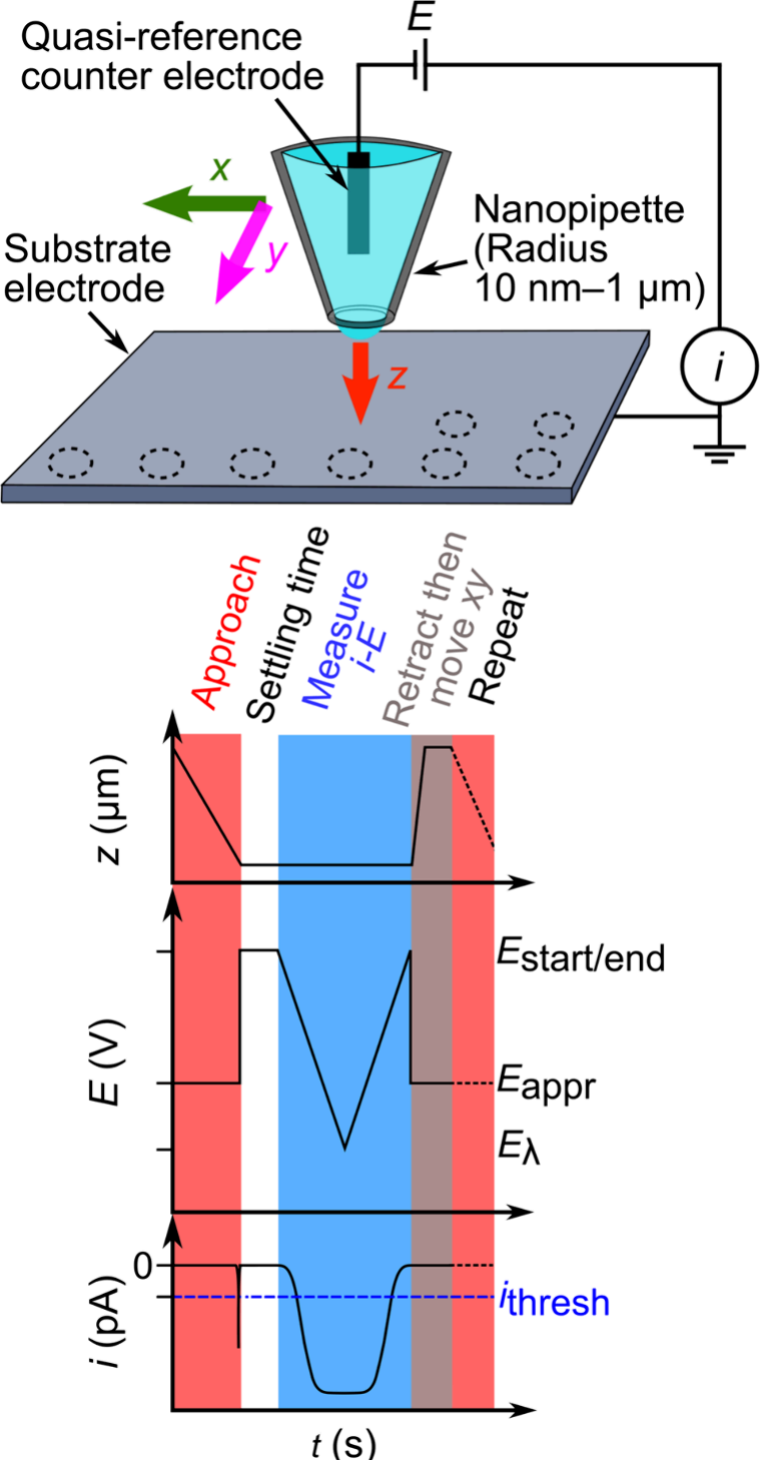
(Top) Schematic of an SECCM voltammetric imaging experiment and (Bottom) the measured (*i*) and controlled (*z*, *E*) quantities during the acquisition of a cyclic voltammogram at a single point in the image.

Scanning electrochemical cell microscopy (SECCM), shown schematically in Figure 1, uses the meniscus at the tip of an electrolyte-filled nanopipette as a mobile electrochemical cell. When the meniscus contacts with an electrode, the current-votlage response measures the electrode’s electrochemical activity in the nanoscale region wetted by the meniscus, while the nanopipette’s vertical position records the electrode’s height. When multiple nanoscale characterizations are performed over an array of points, maps of the electrode’s electrochemical activity and topography are generated.

SECCM imaging has provided insight into a wide variety of applications, a selection of which are described briefly below (see refs (1−9) for further examples). Structure–activity relationships were determined using SECCM to map electrochemical activity and SECCM topographic maps^10^ or complementary techniques (e.g., electron backscatter detection^11,12^ or energy-dispersive X-ray spectroscopy^13^) to map structure. Numerous reaction-substrate combinations have been characterized, such as hydrogen adsorption on polycrystalline palladium,^11^ the hydrogen evolution reaction on silver^12^ and MoS_2_,^10^ and the oxygen reduction reaction on high-entropy alloys.^13^ SECCM has also been used to electrochemically characterize individual nanoparticles to examine particle-to-particle variations, the location of active regions on a single nanoparticle, and the influence of size, shape, porosity, and facets on electrochemical activity.^14^ For example, electrocatalysis at individual gold nanorods,^15^ platinum nanoparticles,^16^ Co_3_O_4_ nanocubes,^17^ and hematite nanorods^18^ has been quantitatively mapped using SECCM. Other behaviors probed with SECCM include the corrosion behavior of metals (see ref (19) and citations therein for details), phase formation, such as the nucleation and growth of gas bubbles^20^ and metals,^21−23^ and charging/discharging of battery electrode materials.^24−26^

The use of SECCM has grown rapidly since its introduction in 2009^27^ and there are many excellent reviews that cover both the operational principles and applications of SECCM.^1−9^ (Note: The term scanning micropipet contact method (SMCM) is used in ref (27) to describe the technique described in this work, which is now commonly referred to as SECCM. SECCM, introduced in ref (28), originally described a measurement with a double-barreled pipette, but that distinction is now rarely made, with SECCM being used to describe experiments with single and dual-barrel pipettes.) A foundational introductory resource that provides education on the fundamentals of SECCM experimentation and is targeted at the novice SECCM experimenter is currently lacking. Without such a resource or the direct support of expert users, navigating the pathway to performing one’s first SECCM measurements presents a significant barrier. This work fills this gap by providing a step-by-step tutorial for performing SECCM experiments, including experiment flowcharts, troubleshooting strategies, photographs, schematics, representative data (of both successful experiments and common problems), videos, a glossary of terms (see Supporting Information, Section S17), and references to original literature. It is directed at individuals with no prior experience of SECCM. The comprehensive steps presented here are written in chronological order for a new SECCM experimenter working with an instrument they do not know or trust. Those with prior experience or access to a fully functioning instrument may choose to skip certain sections. Supporting Information, Section S1 contains suggested pathways for such an experimenter. A reader following the steps described in this work will have learnt how to perform SECCM voltammetric imaging of a model system and the principles and rationale behind each step in this process.

The suggested series of experiments presented herein is based on the cumulative experience gained training individuals within our group and in providing remote assistance to starting SECCM users in other laboratories. Our experience is that individuals following this sequence rapidly gain competence, confidence, and understanding of SECCM measurements, and can adapt and troubleshoot when measurements do not go to plan.

We encourage interested experimenters who successfully complete their first SECCM experiments as described herein to follow up by consulting the recently published *Practical Guidelines for the Use of Scanning Electrochemical Cell Microscopy (SECCM)* presented by Jayamaha et al.^9^ Therein the authors comprehensively review samples, reactions, and types of experiments that have thus far been studied with SECCM, providing sage advice on imaging challenging substrates. We believe the combination of the detailed step-by-step guidelines for performing one’s first SECCM experiments described herein and the guidance on more advanced experimentation presented in Jayamaha et al.^9^ offer novice experimenters a clear pathway toward imaging any sample of interest.

**Figure 2:**
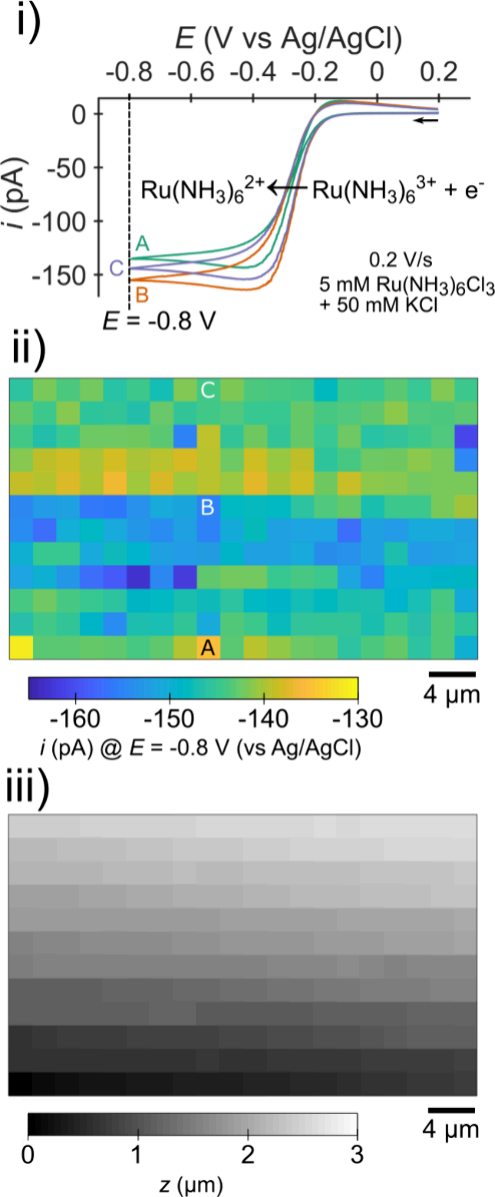
SECCM imaging of Ru(NH_3_)_6_^3+^ reduction on HOPG. (i) Cyclic voltammetry responses at pixels A, B, and C. (ii) SECCM electrochemical map of the measured current, *i*, at *E* = −0.8 V vs Ag/AgCl. (iii) SECCM map of the *z* position of the nanopipette at the surface. The solution in the pipette (pipette radius ≈ 500 nm) was 5 mM Ru(NH_3_)_6_Cl_3_ with 50 mM KCl. The area imaged was 40 × 24 μm^2^ with 20 pixels for *x* and 12 pixels for *y. i*_thresh_ = 3 pA, *E*_approach_ = −0.8 V vs Ag/AgCl, approach speed = 0.5 μm/s, retraction distance = 2 μm, and settling time = 500 ms. Measurements were taken with a Park NX12 (Park Systems, Inc.) instrument using its internal amplifier (1 kHz bandwidth). See Supporting Information, Section S2 for experimental details and Figure S1 for an image of the experimental setup.

The lower portion of Figure 1 shows the vertical position of the nanopipette (*z*), applied potential (*E*), and measured current (*i*) versus time, (*t*) for acquisition of a single cyclic voltammogram during a typical SECCM imaging experiment. The pipette is lowered to the surface (decreasing *z*) with a computer-controlled approach, applying a constant *E* and measuring *i*. A spike in the current occurs when the meniscus contacts the electrode surface, triggering the *z*-movement of the pipette to halt and the vertical position to be recorded. Immediately following surface contact, a short settling time is observed in which the pipette tip position and potential are held constant. After the settling time, the electrochemical measurement (cyclic voltammogram) is acquired with the meniscus in contact with the surface. Upon completion of the electrochemical measurement, the pipette is retracted from the surface and moved to a new *xy* coordinate. This process is repeated for an array of locations on the electrode, allowing cyclic voltammograms and sample height to be measured at each *xy* coordinate. This process generates nanoscale electrochemical and topographic maps, such as shown in Figure 2.

### Suggested System for First SECCM Measurements

The experimental system employed in the generation of Figure 2 represents a system we recommend for first-time SECCM users and that those following the steps described in this work will image for themselves. It consists of highly oriented pyrolytic graphite (HOPG) as a sample electrode, a highly soluble redox mediator (Ru(NH_3_)_6_^3+^ is used for the image, but other mediators with similar characteristics (see below) would also be suitable), a 100–500 nm radius nanopipette, and uses a AgCl coated Ag wire as a quasi-reference counter electrode (full experimental details including a comprehensive list of materials for these and all experiments within this work are given in Supporting Information, section S2). This system possesses uniform electrochemical activity and produces reversible steady-state voltammetry, as can be seen in Figure 2.

We propose this system because the reagents are widely available and do not require synthesis or purification, they are highly soluble and electrochemically well-behaved (fast electron-transfer kinetics). As shown in the topographic image (Figure 2iii), HOPG offers a flat surface. It is renewable by cleavage (*vide infra*), negating the need for complex cleaning or preparation steps, and its moderate hydrophobicity assists with meniscus stability (we recommend beginner users avoid highly hydrophilic samples in their first experiments). SECCM measurements using this combination have been described previously,^29−31^ providing additional data beyond that provided herein to which initial experiments can be compared.

## Evaluating the Instrument Electronics

**Figure 3:**
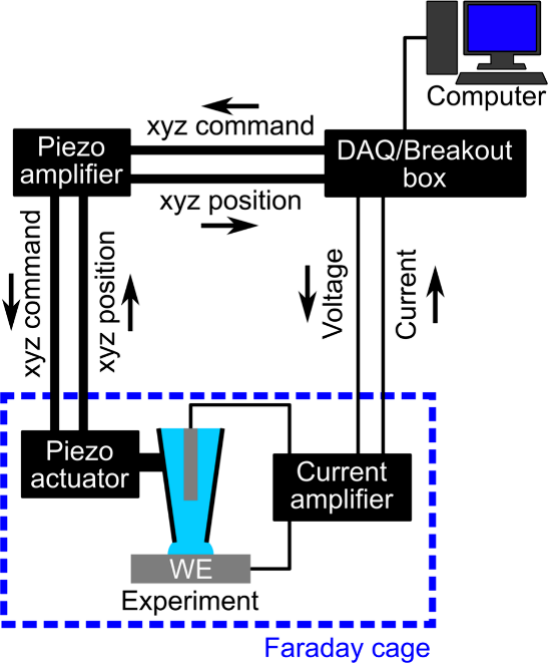
Block diagram of an SECCM instrument, where DAQ is data acquisition and WE is working electrode. Arrows indicate the direction over which information is passed. The thicker wires (xyz command and xyz position wires) represent three individual signals. This diagram is simplified and optional components (e.g., stepper-motors and cameras) are not shown.

SECCM measurements require measurements of low currents (sub-pA to nA range); thus, the current measuring apparatus used must be up to the task and appropriately configured. Figure 3 shows a simplified block diagram of a typical SECCM instrument, where optional components such as stepper motors and cameras (discussed in *Approaching the Surface*) are not shown. The arrows show the direction of signal transmission between the electronic components of the instrument, which are typically encoded as voltages. For example, when the user inputs the desired potential bias for the experiment into the computer, the computer sends this digital signal to the DAQ hardware, which converts it to an analog (voltage) signal. This voltage is received by the current amplifier, which applies the potential bias between the pipette and sample electrodes. The current amplifier converts the typically small (pA to nA) current measured at the sample electrode to a larger voltage based on its gain setting (∼V/pA to V/nA). This voltage is digitized by the DAQ hardware and plotted/stored by the computer.

Piezoelectric actuators (piezos), which are used for fine positioning of the pipette/sample (*vide infra*), require high voltages which are generated by the piezo amplifier. The desired position from the computer is passed to the DAQ hardware where it is converted to a position command (analog or digital voltage). The piezo actuator moves based on the voltage and returns the measured position information (these two values may differ slightly due to the finite time it takes the piezo to move).

The electrical components shown in Figure 3 are connected using wires. We have found short (∼5 cm), unshielded cables offer suitable noise characteristics for wiring the sample (WE) and pipette to the current amplifier when operating inside the Faraday cage. Coaxial BNC cables are sufficient for all other connections passing larger signals (e.g., current amplifier connection to breakout box). The wires and connectors used for the measurements in this work are included in Supporting Information S2.1 List of Experimental Materials.

**Figure 4:**
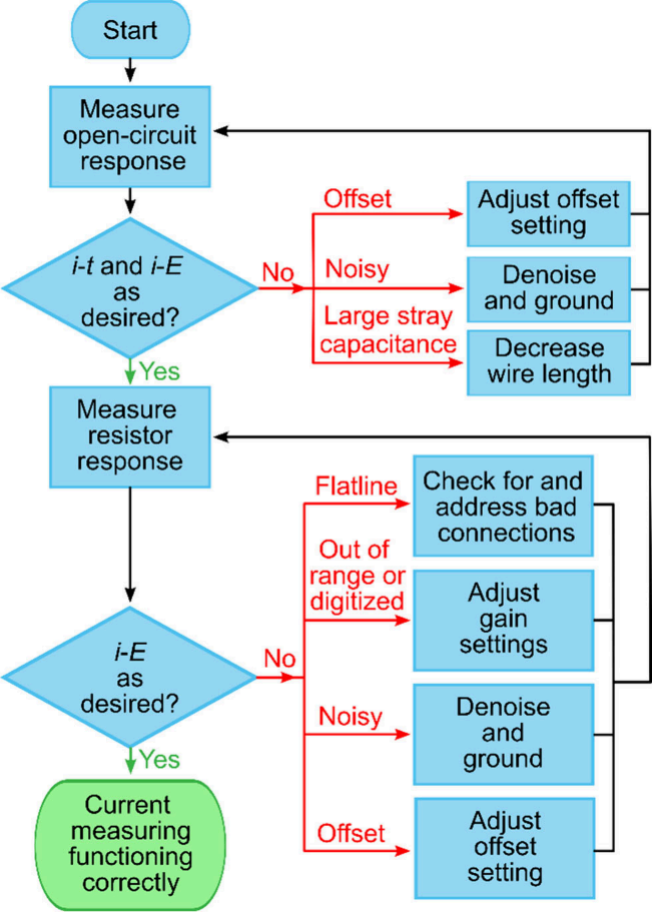
Flowchart showing a systematic approach toward ensuring the SECCM instrument electronics are working as required. Red arrows represent troubleshooting steps used to remedy problems. Each stage is described in detail in the text with representative data provided for each outcome. A printer-friendly full-page version of this flowchart is provided as Figure S16.

This section describes two quick and simple methods to assess the performance of the instrument electronics used in SECCM measurements, with an experiment flowchart (Figure 4) and example data provided. While these steps do not have to be performed before every SECCM experiment, they are an effective way to get to know a new (to the user) instrument or to troubleshoot a misbehaving instrument.

### Open-Circuit Response

As shown in the flowchart in Figure 4, we recommend measuring the open-circuit current response (nothing connected between the leads of the amplifier) as a first step in evaluating whether the instrument electronics are functioning as required. The open-circuit response can inform about the noise and any offset in measuring the current. An offset is a systematic error in measuring a value (i.e., current or voltage) that is either too high or too low (*vide infra*). Given the low current levels in SECCM experiments, it is expected that these and all other electrical/electrochemical measurements described herein are performed within a Faraday cage.^32^ Note, as the circuit is not completed, the value of the applied potential should not influence the current response during open-circuit measurements.

**Figure 5:**
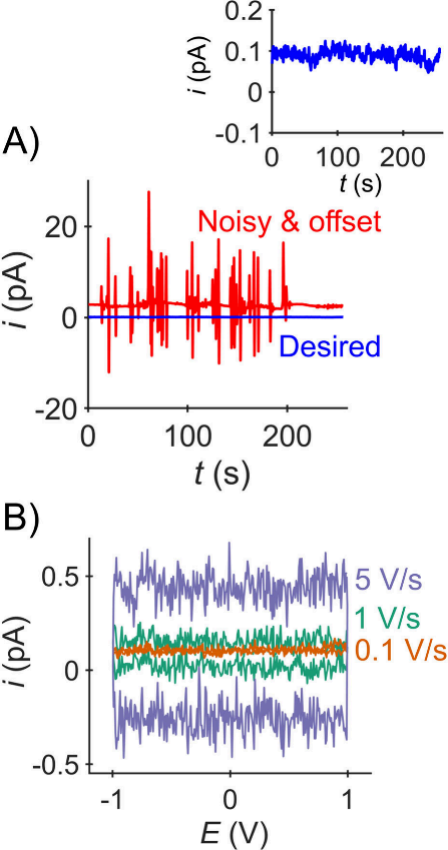
Amplifier responses at open circuit. (A) Open circuit *i*-*t* responses (*E* = 0 V). The inset shows a zoom in of a desirable response in which a small (∼0.1 pA) current offset can be seen. (B) Desirable *i*-*E* response vs scan rate (as labeled). Experimental details in Supporting Information section S2. Park NX12 SICM head internal amplifier (1 kHz bandwidth).

Figure 5A shows *i*-*t* responses of a typical SECCM amplifier (Park NX12 SICM head internal amplifier (1 kHz bandwidth)) at open-circuit, with desirable results in blue and a problematic result in red. The difference between noise levels for the desirable (0.091 pA RMS) and Noisy & Offset curves (4.3 pA RMS) is significant, with low noise allowing reliable operation. The desirable noise levels attained with the Park NX12 SICM head internal amplifier are comparable to those measured with other amplifiers used for SECCM, e.g., FEMTO DDPCA-300 (FEMTO Messtechnik GmbH, Germany) using a 150 Hz bandwidth setting (RMS < 0.25 pA; see Supporting Information, Section S4). The tolerable noise level varies from experiment to experiment (see *Settings for the Computer-Controlled Piezo-Driven Approach* for details), with lower levels preferred. For the experiments described in this work, noise levels <3 pA RMS are likely adequate, but as seen in the blue curve much superior levels may be achieved.

If the noise level is higher than desired and/or contains spikes or periodic signals (e.g., red curve Figure 4A), it suggests interference from environmental sources of noise and/or inappropriate grounding and one should work on denoising and grounding (see refs (32) and (33) for detailed procedures). The noise level is a function of the equipment and its settings (gain, filters), so one should aim for reasonable levels based on one’s equipment and assess performance with like-for-like comparisons. Note that low-pass filters with higher cutoff frequencies let through more noise but allow faster changes to be resolved, so one should consider this balance when considering the experiments being performed (a cutoff frequency ≥1 kHz is adequate for the measurements described herein).^34^

The open-circuit *i*-*t* response also measures the offset in the measured current (i.e., the current reported when zero current is expected). As seen in Figure 5A, the red curve shows an undesirable ∼2 pA offset, whereas the 0.1 pA offset in the blue curve can only be seen in the zoomed-in inset plot. Current offsets arise from the nonidealities of real-world electronic components inside the current amplifier and data acquisition hardware. If a significant offset in the current is measured, the offset setting on the current amplifier (if available) should be adjusted (this might be a via software setting or a physical adjustment, e.g., by turning a screw). Alternatively, one can record the value of the offset and manually subtract it from the data at the end of the SECCM experiment. While it is typically impossible to eliminate offsets completely, minimizing them to values much smaller than the currents you will measure is preferable (we typically take an offset between ±0.5 pA as adequate). A more detailed discussion of the influence of noise and offset on experimental measurements is reserved for the section *Settings for the Computer-Controlled Piezo-Driven Approach* (below).

The open-circuit *i*-*E* response (Figure 5B) is expected to be that of a capacitor (rectangular, centered vertically around zero current; see ref (35) section 1.6 for details). Small current offsets (e.g., ∼0.1 pA in the figure) are of no concern, while larger offsets can be dealt with as discussed above. A nonzero slope is of concern, as it suggests an alternative current path (short circuit) between the two leads, or a problem with the amplifier itself (consult with the amplifier manufacturer/documentation). The capacitive current, *i*_c_, is the absolute distance from the current offset, or equivalently half the distance between the steady currents in the positive and negative scan directions. At open circuit, this capacitance is attributed to the stray capacitance (also called parasitic capacitance), which is the unavoidable capacitance between electronic components simply due to their proximity. The capacitive current, *i*_c_, stray capacitance, *C* (F), and scan rate, ν (V/s) are related by
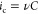
1

As predicted by eq 1, Figure 4B shows proportionally larger *i*_c_ (separation between the currents on the forward and backward scans) at higher scan rates. We estimate the capacitance from the 5 V/s scan as *C* ≈ 0.3 pA/5 V/s = 60 fF. A linear fit of *i*_c_ vs *v* (Figure S5) gives a more accurate estimate of ∼70 fF.

While stray capacitance is unavoidable and is typically larger than the double-layer capacitance of a region probed by SECCM,^36^ we desire to minimize its value to avoid capacitive currents dominating measurements. Assessment of data from our lab and the literature suggests that a value of <15 pF is achievable by most experimenters.^37−39^ For the measurements described herein, even a value of 15 pF (giving *i*_c_= 3 pA at 0.2 V/s) would be tolerable compared to the ∼100 pA current (*vide infra*), but as shown above, much better is achievable. Excessively long unshielded leads connecting to the amplifier can cause a high stray capacitance. We recommend using short (∼5 cm) leads, which may necessitate relocating the amplifier.

### Resistor Response

A resistor provides a reliable electrical response as described by Ohm’s law
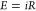
2where *E* is the potential, *i* is the current and *R* is the resistance. As such, connecting a resistor in place of the pipette/sample (see Figure S6 for images of example experimental setups), and characterizing its *i-E* response is a fast, simple, and effective method to characterize the performance of the current measuring and data acquisition components of an instrument. It is recommended to use a resistor that will deliver currents of a comparable range to those anticipated in experiments (∼pA-nA; see *Imaging Parameters* for discussion of predicted current). Resistors in the 50–1000 MΩ range are readily available and are appropriate in most cases. Whether beginning a new SECCM experiment, benchmarking the performance of a new instrument, after the addition of a new experimental component, troubleshooting a misbehaving experiment, or familiarizing a user with a new instrument/software, measuring a resistor is a quick way to determine whether the electrical side of a measurement is performing as required.

**Figure 6:**
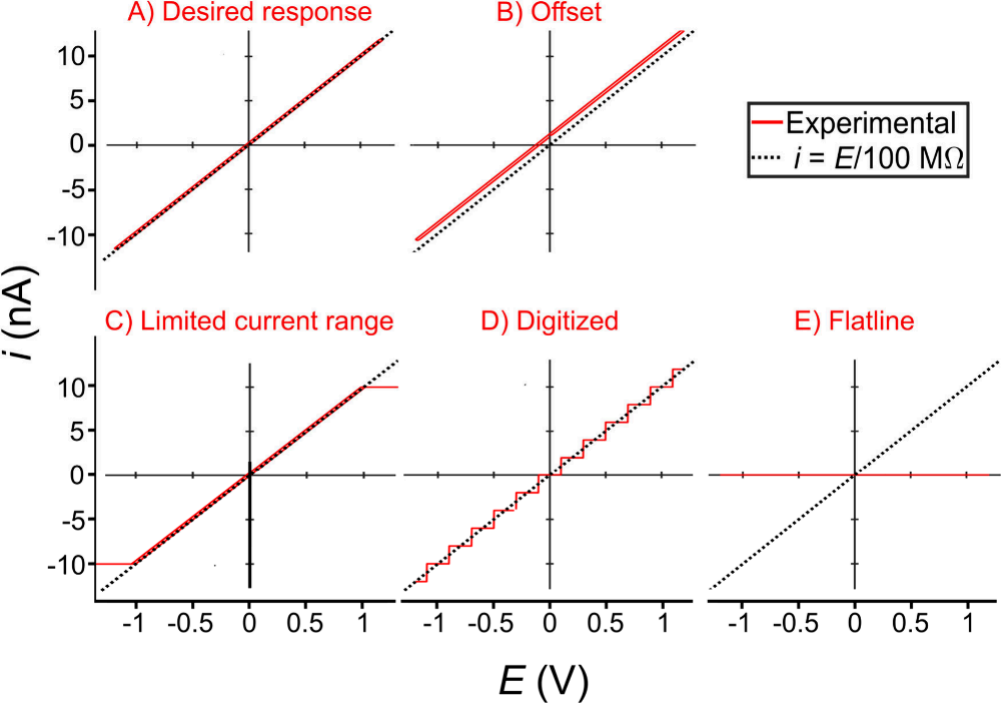
Possible *i*-*E* responses for the voltammetric characterization of a 100 MΩ resistor (solid) compared to the expected response as predicted by eq 2 (dashed). A home-built SECCM instrument with a DDPCA-300 (FEMTO Messtechnik GmbH, Germany) amplifier with 150 Hz bandwidth setting was used for measurements. See Supporting Information, sections S2 and S6 for more experimental setup details. Scan rate: 0.2 V/s.

Measuring a resistor allows one to check the noise level, gain settings, offsets, and electrical connectivity. After connecting the resistor to the instrument (the direction of connection does not matter), one can perform *i-E* measurements to evaluate the instrument’s current-measuring components. Figure 6 shows common responses to the voltammetric characterization of a 100 MΩ resistor. Figure 6A shows the desired linear response (red) with an intercept of 0 nA and a slope of 1/*R* (∼10 nA/1 V = 1 × 10^–8^ A/V = 1/100 MΩ), as is predicted by eq 2. The solution to eq 2 is shown by the dashed line in Figure 6. Note: resistors are manufactured with specified tolerances that are typically in the range of 1–5%, thus a small difference in the slope from that predicted is to be expected. See Figure S7 for a zoomed-in view of the desired response in Figure 6 to see the small difference (∼0.6%; less than the 1% tolerance) in the measured slope compared to the solution to eq 2. If the slope differs significantly from that expected, this may be a symptom that the gain on the current amplifier and/or in the software have dissimilar values or that the resistor being used has a different resistance value to that used in calculations.

An *i*-*E* response that does not pass through the origin, such as Figure 6B, indicates an offset. As discussed in *Open-Circuit Response*, offsets can arise from any of the components (see Figure 3), from the current amplifier, the potential source within the amplifier, or the signal controlling the current amplifier (e.g., DAQ). As such, the offset seen in Figure 6B could be in the applied potential, the measured current, or a combination of the two. For example, the data shown are compatible with either a 1 nA or −0.1 V offset. An *i-E* measurement at open circuit (see above) can be used to determine the current offset alone, with any remaining offset being due to a potential offset. Depending on the amplifier used and software options, it might be possible to adjust the current and/or voltage offsets with adjustment screws on the amplifier and/or via software. Alternatively, the offset values may be recorded and manually subtracted from data after experiments are performed. Depending on the source of the offset and the design of the amplifier/data acquisition system, the offset may be consistent between different amplifier gains or vary between them and stable/unstable with time; this should be determined experimentally.

Undesirable responses may occur if the current gain is too high or too low for the signal to be measured. As shown in Figure 6C, a gain setting that is too high gives a current response that swings between flat responses corresponding to the maximum and minimum measurable current levels with the portion around *E* = 0 V agreeing with the ideal response. In this measurement the current limits were ±10 nA, while the expected maximum/minimum current for the ±1.35 V range calculated through Ohm’s law is ±13.5 nA (=±1.35 V/100 MΩ). Choosing a lower gain level can remedy this problem. Alternatively, if the gain setting on the current amplifier is too low, a digitized response like that shown in Figure 6D might be seen due to the limited precision to which the DAQ can measure potentials. If a digitized result is observed, the gain setting should be increased.

If a flatline response like that shown in Figure 6E is recorded, first check that the current range on the graph is appropriate and that a response is not hidden by an incorrect y-scale. A truly flat *i-E* response is indicative of incorrect connections, broken wires, and/or corroded connectors. A visual check for any obvious issues is a good first step, but it is crucial to use a multimeter to systematically check connectivity, that an appropriate voltage is being applied, and, in the case of an external amplifier, that a nonzero output is coming from the “measured current” output. A noisy response might also occur (not shown), and in this case one should consult refs (32) and (33) for information on denoising and grounding.

Erroneous responses may also be caused by incorrect use of software. Typical problems include differences between the settings on the amplifier and those in the software, the selection of input/output channels being inconsistent with the experimenter’s expectations, or incorrect parameters for electrochemical experiments. Given the diverse range of software, we direct the experimenter to the documentation for their software and/or suggest the use of a multimeter (voltage source) to measure outputs (check inputs) and compare to expectations.

Note that a single measurement may contain multiple undesirable features. In this case, each should be addressed in turn and the tests repeated until a desirable response comparable to that shown in Figure 6A is obtained.

## Pipettes

**Figure 7:**
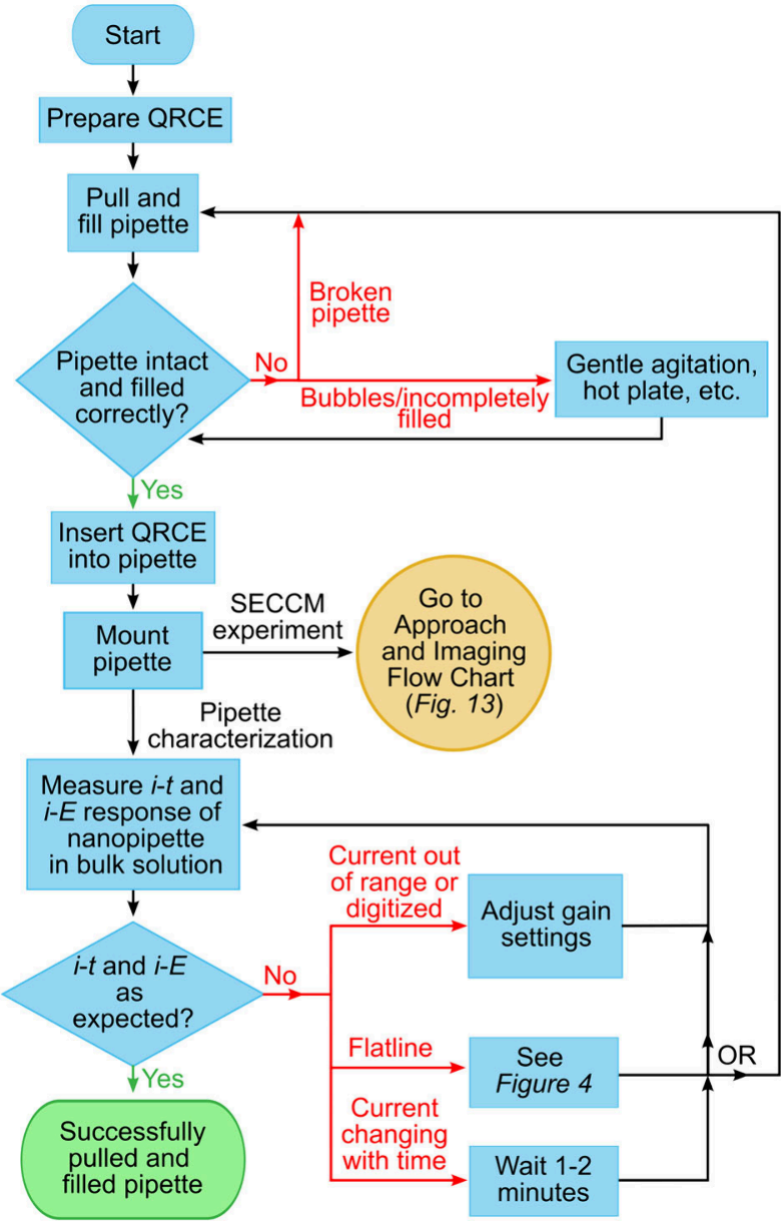
Pipette preparation flowchart. A printer-friendly full-page version of this flowchart is provided as Figure S17.

SECCM experiments utilize nanopipette probes as consumable one-use items. NB: Within the field, the terms nanopipette, pipette, probe, tip, and combinations thereof are used interchangeably. The preparation (pulling, filling, characterization) of nanopipette probes is typically performed on an as-needed basis immediately before experiments and is described in the following subsections. A flowchart for the preparation of nanopipette probes is shown in Figure 7 with the steps and troubleshooting strategies described in the following subsections. At the branch in the flowchart from the *Mount pipette* box, one can either follow the *Pipette characterization* path or continue toward completing an *SECCM experiment* by jumping to the eponymous section of this work. We suggest those preparing pipettes for the first time begin by following the *Pipette characterization* path, which provides rapid feedback on this key experimental skill. Note: following this path will leave the pipette unsuitable for SECCM experiments.

### Pulling Pipettes

Nanopipettes are prepared using commercially available glass (borosilicate or quartz) capillary tubing and pipette pullers. Either material is appropriate, but we recommend using capillaries with thin glass filaments inside them (see Supporting Information section S2), as the filament aids in the complete filling to the nanopipette tip by capillary action. Pipette pullers heat the glass with a resistive coil or a CO_2_ laser and then separate it into two parts by pulling (a CO_2_ laser provides the higher heat necessary to melt quartz). Nanopipettes are fabricated to one’s desired dimensions by manipulating the heating and pulling parameters. We recommend first-time SECCM experimentalists pull pipettes to a tip radius of 100–500 nm, as these are easier to fill and are likely to give relatively large currents (∼25–200 pA) with typical 5 mM concentrations. The reader is encouraged to read the user manual of their pipette puller to learn its controls and is directed to Shae et al. for a review of general nanopipette fabrication,^40^ Zhou et al. for a review of the fabrication, characterization, and filling of sub-10 nm nanopipettes,^41^ and Jayamaha et al. for a summary of pulling parameters documented for SECCM pipette fabrication and resulting pipette tip size.^9^ Note that pipette pullers are not all identical, and performance can change over time (heating coils can oxidize, laser power can degrade, laser puller retro-reflective mirrors become dirty, etc.), so one should be cautious in blindly following a pulling recipe.

### Filling Pipettes

Filling nanopipettes to their very tip without bubbles or voids, is necessary to provide good electrical connectivity. The small diameter of nanopipettes makes filling them completely one of the more challenging tasks for first-time SECCM experimentalists. In training sessions completed in our lab, new SECCM experimenters only successfully filled 2–3 pipettes out of 10 on average. Do not be discouraged, with practice and a rapid method of assessing filling success (*vide infra*), a novice can become adept in a handful of hours and confidently achieve success rates of >60% within their first 3 days of experiments. We encourage those without experience filling pipettes to examine their pipettes under an optical microscope to check for bubbles and pipette breakage (*vide infra*), but an experienced experimentalist with good eyesight can learn to spot these problems without a microscope. In this section, we discuss recommended methods for nanopipette filling and provide references detailing alternative methods.

**Figure 8:**
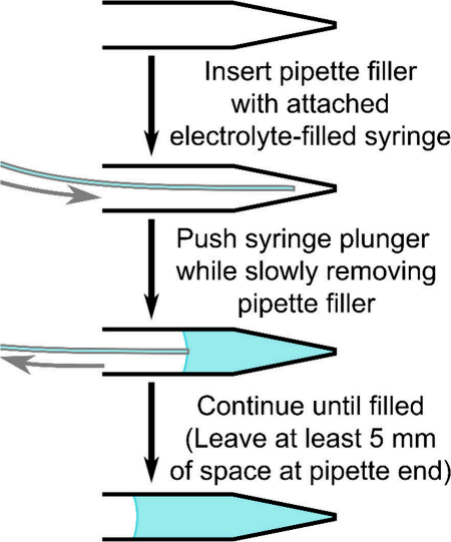
Schematic of the backloading pipette filling method steps. Schematic inspired by Simonis and Hennig.^42^

We recommend the backloading method (shown schematically in Figure 8) for filling nanopipettes. In this method, a thin flexible needle (MicroFil pipette filler, WPI) attached to the end of an electrolyte-filled syringe is inserted into the back of the nanopipette. The tip of the pipette filler is then positioned at a point near where the tapering of the pipette begins, and the syringe plunger is pushed with a steady pressure while slowly removing the pipette filler from the tip to fill the nanopipette with solution. As capillary action is a predominant force in pipette filling, the angle it is held is unimportant, with angles from vertical to horizontal chosen based on experimenter preference. The exact fill level is not a critical parameter; however, it should be sufficient that the reference electrode well contacts the solution while leaving at least 5 mm of space at the top to prevent the solution from being pushed out the end of the pipette when a reference electrode is inserted; we suggest aiming for a half-full pipette. A video showing a pipette being filled via backloading is provided as Supporting Information. Tip: cutting the pipette filler to a shorter length with sharp scissors or a razor blade, leaving sufficient length to reach the end of the pipette, minimizes the vibrations in its tip making it a little easier to insert into the end of the pipette.

It is important to note that the nanopipettes are fragile, so one must take care when manipulating the pipette to not brush against the tip end. Note, when stored in the laboratory environment pipettes can become contaminated with adventitious organic contaminants making them hydrophobic and harder to fill, so it is good practice to use freshly pulled pipettes (<2 h). In normal circumstances, we find it unnecessary to filter the solutions used for SECCM experiments; however, if particulates in the solution are of concern, a syringe filter can be used between the syringe and pipette filler. Note, if evaporation of the solution is a concern, for example, if an experiment is planned for an extended period (>2 h), a layer of mineral oil may be added to the top of the nanopipette;^10^ however, we do not recommend this additional step in the introductory experiments described herein.

**Figure 9:**
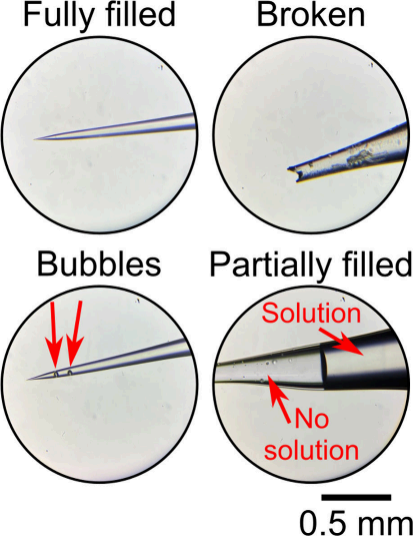
Optical micrographs of the possible outcomes of attempting to fill a nanopipette. Magnification factor of objective lens is 10× and magnification factor of the eyepiece lens is 10×. Further examples micrographs are available in Supporting Information section S8. Pipette radius ∼500 nm.

Figure 9 shows optical micrographs of commonly observed outcomes when attempting to fill nanopipettes. The top left optical micrograph shows an intact nanopipette where the tip is filled without voids or bubbles, as required for SECCM experiments. While the tip of the ∼500 nm radius nanopipette cannot be resolved with optical microscopy due to the diffraction limit, the nanopipette tapers to a point when viewed side on, as shown in Figure 9 (top left). However, if the pipette end looks jagged, as shown in Figure 9 (top right), the tip is broken, and a new pipette must be prepared. While pipette breakage can usually be seen with an optical microscope, those closer to the pipette end will not be as obvious as that shown in this figure (see Figure S8 for examples of pipettes broken close to the tip).

During the filling process, bubbles are often generated within the pipette (typically due to erratic movement of the pipette or syringe plunger). Figure 9 (bottom left) shows a pipette with two bubbles adhered to the pipette wall. Bubbles within the nanopipette affect electrolyte transport^43^ and the current measured in the experiment. Sometimes bubbles in a pipette can be seen easily with the naked eye; however, this is not always the case. If a pipette contains bubbles, it may be possible to remove them. Methods for doing so include reinserting a pipette filler and withdrawing the syringe plunger to extract the bubbles or using gentle agitation (e.g., by flicking with a finger or rubbing with serrated tweezers/threads of a screw) to attempt to release the bubbles mechanically. See the Supporting Information for videos of gentle agitation with tweezers and a screw. Nanopipette tips are fragile, so one should take care not to break the tip when using these methods. We direct the reader to Jayamaha et al. for a description of two other methods for removing bubbles: negative pressure and centrifugal force.^9^

Another common problem encountered when attempting to fill pipettes is partial filling, with the solution failing to reach the pipette tip, as shown in Figure 9 (bottom right). As discussed above, pulling nanopipettes from glass capillaries containing a glass filament can aid in filling completely to the pipette tip. We find that using the gentle agitation methods described above can usually fix partial filling problems for >100 nm tip radius pipettes. On the rare occasions gentle agitation does not fix the problem, we typically discard the pipette and fill a new one, but other methods have been reported to aid in complete filling such as a thermally driven method,^44^ dynamic microdistillation,^45^ microcentrifugation,^46^ and microwave radiation-induced heating.^47^ We suggest the reader consult these references for details on these methods.

While not performed in this work, it is possible to functionalize the outer surface of the pipette with hydrophobic molecules or other surface coatings to alter the pipette’s surface properties. Typically, a silanization agent reacts with the hydrophilic –OH groups on the glass surface replacing them with hydrophobic moieties. This process, termed *silanization*, renders the outer surface of the pipette hydrophobic and prevents the aqueous electrolyte from creeping up the outer walls of the pipette.^48,49^ While solution on the outside of the pipette is not expected to affect the current in ideal SECCM measurements, it may cause the formation of electrolyte crystals or encourage the meniscus to spread far beyond the pipette orifice, both of which may be detrimental to SECCM imaging. Silanization of the outer wall of the pipette is recommended during the imaging of hydrophilic samples to keep the area wetted by the droplet stable. However, using a hydrophobic sample (HOPG) for one’s first experiments as we recommend herein renders silanization unnecessary. We direct those ready to move on to their own samples to Jayamaha et al. for a more detailed discussion on pipette functionalization and the silanization procedure.^9^

### Quasi-reference Counter Electrodes (QRCEs)

A quasi-reference counter electrode (QRCE) inside the pipette provides the reference against which the electrochemical behavior of the electrode is measured. In a typical SECCM experiment, a QRCE is inserted into the barrel of the electrolyte-filled pipette until its end is 1–3 cm from the end of the pipette tip. A Ag/AgCl wire quasi-reference/counter electrode is the most commonly used internal electrode, due to its robustness, ease of fabrication of a suitable size to insert into a pipette, and stability in confined electrochemical environments (for a detailed discussion on the robustness and stability of Ag/AgCl QRCEs see ref (50)). As such, we suggest the Ag/AgCl wire as a QRCE for first-time SECCM experimentalists. Note, Ag/AgCl QRCEs are not compatible with all experiments (see ref (9) for a summary of alternative QRCEs used in SECCM experiments).

A Ag/AgCl QRCE can be made by submerging a silver wire in a sodium hypochlorite bleach solution (e.g., Chlorox) or applying an oxidizing potential in a chloride-containing solution.^50,51^ The electrically insulating AgCl should be removed from the top of the wire with sandpaper or a blade to expose bare Ag and ensure good electrical contact. When inserting the electrode into the pipette, the wire should be straight and should not scrape the pipette’s edges. Doing so lessens the chance of the AgCl coating detaching and clogging the pipette or interfering with any electrochemical measurements.^50^ Once inserted, the wire is bent over the end of the pipette to stabilize its position.

### Mounting the Pipette

**Figure 10:**
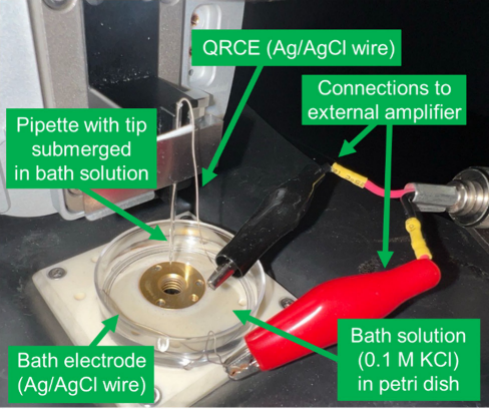
Experimental setup for measuring the resistance of a nanopipette in an electrolyte bath.

Once the QRCE has been inserted into the pipette, the pipette should be mounted into the instrument, secured using a screw and washer or clip, and the exposed end of the QRCE should be connected to the instrumental amplifier (typically through an alligator clip). When operating inside a Faraday cage, unshielded wires perform adequately for connecting the pipette to the amplifier when the total length of the leads (including alligator clips) is kept short (∼5 cm). Such lightweight wires assist in avoiding applying undue stress to the pipette and risking snapping it. Again, avoid touching the tip end of the pipette at any point. An image of a pipette secured by a clip with the Ag/AgCl reference electrode connected to the amplifier is shown in Figure 10 (a zoomed-in view of the pipette with inserted and connected QRCE can be seen in Figure S9 of the Supporting Information; another example is shown in Figure 14 below).

Given the dexterity required to load pipettes into the instrument without damaging them, we recommend first-time SECCM experimentalists practice loading unfilled *dummy pipettes* into the instrument. In addition to practicing the required skills, this allows one to make sure wires are appropriately positioned.

### Pipette Characterization

A nanopipette’s size and shape (typically described by the aperture size, half-angle, and glass thickness) influence the response in scanning probe microscopy experiments;^27,52^ therefore, nanopipette characterization is an important step in preparing for SECCM experiments.^53,54^ In addition to optical microscopy (described above), nanopipettes are commonly characterized using methods such as electron microscopy,^55−60^ the electrical resistance of the pipette^61^ (see below), and their combinations. Other reported characterization methods include atomic force microscopy,^62,63^ meniscus velocity,^64,65^ bubble pressure,^66^ and quasi-controlled breakage.^67^ See refs (41), (53) and (54) for a detailed discussion of nanopipette characterization methods.

#### Measuring the *i*-*t* and *i*-*E* Response of a Nanopipette in Bulk Solution

As described above, the dexterity and coordinated motion required to fill nanopipettes is one of the most challenging steps for beginning SECCM experimenters. While reading descriptions and watching videos can help in learning this skill, direct experience with rapid feedback is essential to quickly develop confidence and competence. Optical microscopy, as described in *Filling Pipettes*, is our recommended first step to assess the preparation of a nanopipette. This can then be followed by quickly measuring the electrical resistance of the pipette, as described in this section. Electrical characterization both provides feedback on the pipette-filling technique and measures the resistance of a nanopipette, which can be used to determine its size (*vide infra*). Note that these electrical measurements will leave pipettes with electrolyte residue on their outside, which is incompatible with SECCM measurements. Nonetheless, we find such procedures invaluable in providing the rapid feedback needed to deliver confidence and competence in pipette filling, and to inform on the distribution of typical pipette resistances.

Electrical characterization measures the solution resistance between the electrode in the pipette and one in a bath of electrolyte, using an experimental setup such as shown in Figure 10. As current is ionic, no electroactive species is necessary, while using a high ionic strength filling solution (e.g., 0.1 M KCl) avoids the influence of the surface charge on the glass on the current response.^68,69^ Interpretation is further simplified by using the same electrolyte in the bath and pipette with identical QRCE electrodes.

**Figure 11:**
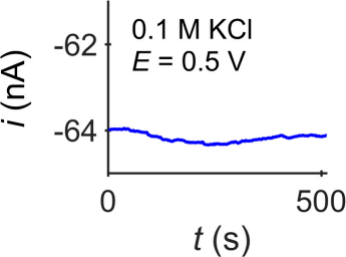
A desirable (stable) *i*-*t* response of a nanopipette filled with 0.1 M KCl in a bath of 0.1 M KCl. Ag/AgCl wires served as both the QRCE in the pipette and the bath electrode. Measurements acquired using a Park NX12 instrument with an external DDPCA-300 (FEMTO Messtechnik GmbH, Germany) amplifier operating with a 400 Hz bandwidth and 10^7^ V/A gain. For more experimental details, see Supporting Information, section S2.

The ion current passing through the pipette tip opening can be measured by applying a voltage between the two electrodes. As shown in Figure 11, a desired current–time response at a constant applied potential is constant (<1% variation over 500 s). A current response that is changing with time could be indicative of bubbles moving/dissolving in the pipette, salt crystals dissolving, or particulate contamination. An unstable response that stabilizes when remeasured after a few minutes indicates the pipette was likely well-filled, whereas continued unstable measurements indicate problems with the filling. If a flatline (zero current) response is observed one should follow the procedures described in *Resistor Response* to determine whether the problem lies in the electrical (or pipette) portion of the experimental setup. If the current response indicates an incorrect current range (out of range/digitized response), one should adjust the gain setting on the amplifier and repeat the measurement (see Figure 6 and discussion thereof for details and examples).

**Figure 12:**
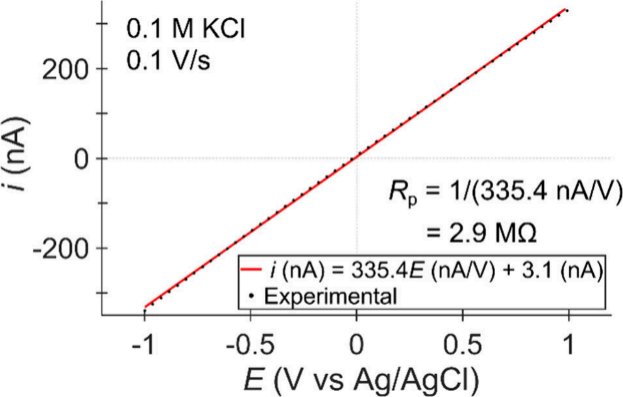
*i-E* response of a nanopipette filled with 0.1 M KCl in a bath of 0.1 M KCl. The red line shows the line of best fit with the equation shown in the legend. Ag/AgCl wires served as the QRCEs in both the pipette and the bath electrode. Measurements were acquired using a Park NX12 instrument with external DDPCA-300 (FEMTO Messtechnik GmbH, Germany) amplifier operating with a 400 Hz bandwidth and 10^7^ V/A gain. For more experimental details, see Supporting Information, section S2.

The *i*-*E* curve for a nanopipette filled with 0.1 M KCl submerged in a 0.1 M KCl bath is shown in Figure 12 (black points) with best-fit line (red). The resistance of the pipette, *R*_p_, can be determined by slope = 1/*R*_p_. For this pipette, the 33.54 nA/V slope gives *R*_p_ = 2.9 MΩ. *R*_p_ is related to the pipette geometry by^54,70,71^
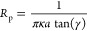
3where κ is the solution conductivity (assumed to be equal in the bath and pipette, *vide supra*), *a* is the pipette tip radius, and γ is the half-angle of the pipette.

Rearranging eq 3 using the measured pipette resistance and conductivity of 0.1 M KCl (∼1.29 S/m)^72^ and a half-angle of γ = 12° estimated from optical microscopy suggests the pipette shown in Figure 12 has a radius of approximately
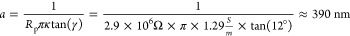
4

Common γ values reported in literature range from 5 to 15°.^1,27,53,73^ Solution conductivity can be determined experimentally using a conductivity meter or by searching literature such as reference (72). Pipettes pulled from the same glass with the same parameters and the same fill solution should have similar resistances (typically ±60%). As such, consistent resistances are a useful proxy for the success of the filling procedure.

## SECCM Experiment

**Figure 13:**
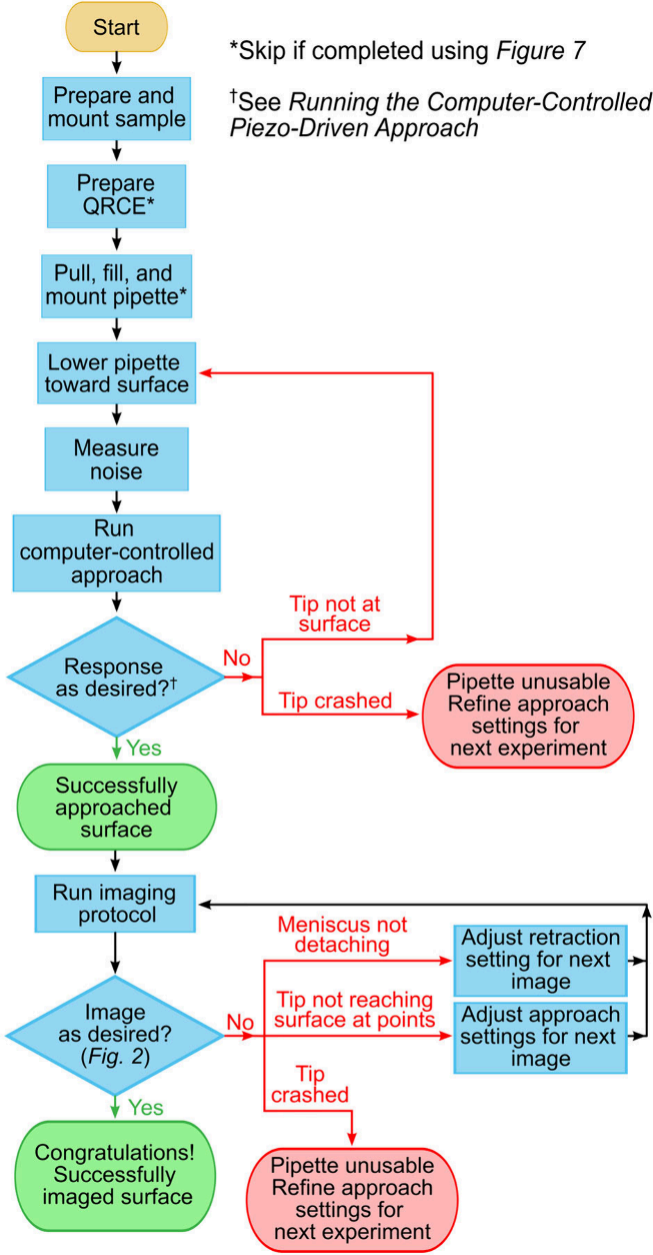
SECCM approach and imaging flowchart. A printer-friendly full-page version of this flowchart is provided as Figure S18.

If the *SECCM experiment* branch of the pipette preparation flowchart (Figure 7) is chosen, one should then follow the steps leading to obtaining an SECCM image as shown in Figure 13 and described in this section. The first step is to *prepare and mount sample*, which as the flowchart suggests, should be performed before preparing the pipette. NB: Within the field the terms substrate, sample, surface, and electrode are used interchangeably. While a broad range of electrodes are suitable for SECCM imaging,^2,4^ for the reasons detailed in *Suggested System for First SECCM Measurements*, we recommend first-time SECCM experimentalists use HOPG.

### Sample Preparation and Mounting

To aid in sample handling and achieving a reliable electrical connection, it is recommended to mount the sample onto a durable conductive base; for this purpose, we choose to use an inexpensive 20 mm diameter metallic specimen disk (Ted Pella) (other flat, conductive, durable surfaces would be appropriate). Affixing the sample to the support using a conductive adhesive such as carbon conductive tabs, copper tape, and/or conductive glue/paint both secures the sample and allows electrical contact to be made through the support. Note that sufficient time should be taken to allow mounting adhesive materials to cure as they will typically shrink during this period causing the sample to be a “moving target” during imaging. It is good practice to check for conductivity from the top of the sample to the support with a multimeter (expected resistance for good electrical connection to HOPG < 10 Ω).

**Figure 14:**
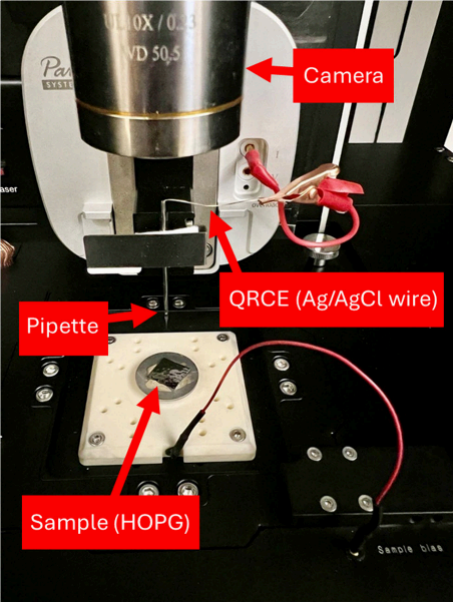
Image of an SECCM experiment setup for the approach to and imaging of HOPG. QRCE = quasi-reference counter electrode. See Supporting Information section S9 for zoomed-in views of the pipette and sample regions of this image. Other experimental setups are shown in Supporting Information section S3.

Alligator clips and wires can then be used to connect the sample to the instrument. Note, alligator clips may corrode, so it is good practice to clean them with sandpaper to ensure a good connection. An example of a HOPG sample mounted on a metal disc using silver paint and connected using a red wire is shown in Figure 14 (see Figure S9 for a zoomed-in image of the connected sample). Alternative experimental setups are shown in the Supporting Information, section S3. Immediately before the experiment, a clean HOPG surface should be exposed; this can be rapidly achieved using the *scotch tape cleavage method*, and the reader is directed to the SI of ref (74) for detailed instructions.

### Approaching the Surface

The process of approaching the SECCM pipette tip to the sample surface, from where electrochemical characterization and imaging can be performed, is outlined in the flowchart shown in Figure 13 and described in detail in this and the subsequent sections. Control of the vertical position of the pipette is achieved by coupling the computer-controlled fine positioning of a piezoelectric actuator (piezo) to long-range experimenter-controlled coarse positioning actuators. Piezos used for SECCM have a limited expansion range and high resolution (typically 10–200 μm range and <1 nm resolution) while coarse-positioning actuators (stepper motors, micrometers, or fine-pitch screws) typically have ∼1–10 cm range and ∼0.5–10 μm resolution. The strategy is to begin by using coarse positioning system to set the pipette at such a height that the piezoelectric actuator can then controllably touch the meniscus to the sample.

#### Lowering the Pipette toward the Surface (Coarse Positioning)

Once the pipette is filled and mounted to the instrument, the QRCE loaded, the sample prepared and mounted, and all electrical connections made, the experimenter should use the coarse positioning actuators to lower the pipette to a position close to the surface, but without contacting it. If the pipette touches the surface in an uncontrolled manner, it will break. An ideal initial pipette position is within the range of the piezoelectric positioner from the surface (∼100 μm from surface). Reliably achieving this on the first attempt takes practice; in our experience, experimenters typically gain the necessary expertise in ∼5–10 attempts.

Depending on the instrument setup, coarse positioning may be performed by hand with a micrometer or fine-positioning screw or by using a stepper motor, which is controlled in software or by an external controller. The approximate pipette-sample separation is visualized optically using a side- or top-view camera/microscope equipped with a long working distance objective or viewing from the side by eye, possibly aided by a magnifying glass. An experimental setup with a top-view camera is shown in Figure 14 and side-view camera setups are shown in Figures S2 and S3. Appropriate lighting, camera position, focus, and angle are important to achieving an in-focus image of the end of the pipette and we encourage experimentation with a dummy pipette to adjust these to get informative images. NB: Adding electronics (cameras, light source, etc.) inside the Faraday cage can introduce a source of electromagnetic noise; this should be assessed using the procedures described in *Evaluating the Instrument Electronics*. If additional noise is observed, assess if it might be eliminated through switching off, unplugging, and/or appropriate grounding of the components, otherwise, they may need to be removed from the Faraday cage after coarse positioning is completed.

If using a reflective sample, such as HOPG, one has the advantage of seeing the pipette reflection as the pipette tip gets close to the surface (see Figure S10). When using a top-view camera, determining the distance between the focal planes when focusing on the sample and the tip provides their separation (see Figure S11). In each case, moving the pipette a known distance (either using piezo or coarse positioners) and comparing before and after the images can provide a sense of scale.

We observe that positioning the pipette within 100 μm of the sample surface, a distance comparable to the width of a human hair, presents a challenge to many starting SECCM experimenters who may be unfamiliar with such short length scales. Positioning at >1 mm is common on first attempts, which leads to unnecessarily long approaches to the surface. However, those initially struggling with this step should not be disheartened, by relating the observed starting position to the distance from the sample determined through the approach (see *Running the Computer-Controlled Piezo-Driven Approach*) a familiarity with length scales involved, and rapid improvement, can be achieved. Our observations are that a typical beginning experimenter can consistently position the pipette tip ∼100 μm away within 5–10 experiments.

During coarse positioning, be careful and move the pipette slowly to avoid crashing the pipette tip into the surface. Any possible damage can be assessed by optical microscopy, with a broken pipette being unusable, and requiring repetition of the *Pulling pipettes* and *Filling pipettes* stages. Once the pipette is near the surface, move on to the computer-controlled approach protocol.

#### Settings for the Computer-Controlled Piezo-Driven Approach

The computer-controlled approach, which was shown in Figure 1 and described in the discussion thereof, sees the pipette moved toward the surface at a constant speed while a potential is applied between the sample and the QRCE in the pipette. When a nonzero current (above an experimenter-specified threshold level) is detected, the motion is halted. A successful completion of this procedure sees the meniscus controllably contact the sample without pipette damage. Success requires careful selection of three major parameters: the voltage during the approach, approach speed, and current threshold (parameter names may vary from instrument to instrument). We suggest first-time SECCM experimentalists start with conservative approach settings, as described below. Once reliable experiments are achieved, one can optimize parameters for speed while maintaining stability.

The bias between the sample and QRCE as the pipette is approached to the surface, *E*_appr_, determines which electrochemical process(es) will occur when the circuit is completed, i.e., when the meniscus makes contact. As such, an appropriate approach voltage setting depends on the redox reaction(s) in the experiment, the sample electrode, and the QRCE employed. In the first suggested experiments described in this work, which employ a soluble redox species with fast kinetics, we suggest that the experimenter pick an approach voltage where the analyte will be oxidized or reduced at a transport-limited rate. This can be achieved with an approach potential >0.1 V beyond *E*^0^ (measured vs the internal QRCE). To determine an appropriate approach voltage one can search literature for previous work using the chosen reaction, consult a reference table of standard electrode potentials in a textbook such as ref (35), or perform conventional electrochemical measurements of the system. Such a large overpotential will result in a relatively large current upon meniscus contact, which is most readily detected. More generally, the approach potential is chosen such that a detectable current will be observed but that no undesirable electrochemical processes will occur, as discussed in more detail below.

The approach speed (μm/s) sets how quickly the z-piezo extension increases and thus how quickly the pipette approaches the sample. While faster approach speeds will decrease the duration of an SECCM experiment, too high an approach speed increases the risk of crashing the pipette into the sample due to the finite time to halt the pipette motion upon meniscus contact. The response time arises from the instrumental components in the chain connecting the current measurement, through threshold detection, to the cessation of piezo movement. While appropriate approach speeds will depend on the instrumentation used and experiment performed, we find initial values of 0.2 – 0.5 μm/s to work on a range of instruments for the pipette sizes described herein.

**Figure 15:**
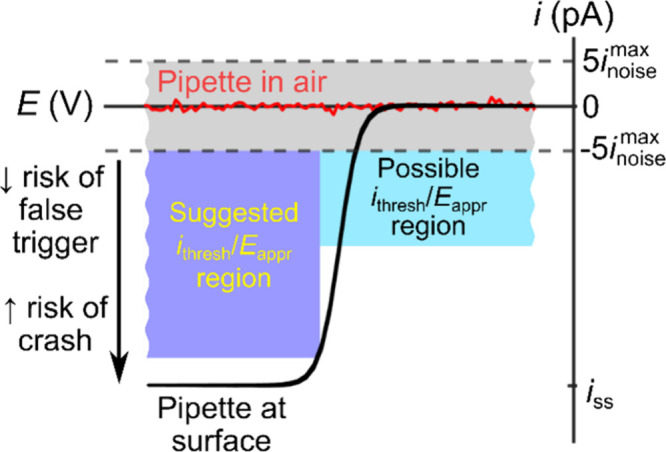
Schematic of an *i*-*E* response of a pipette suspended in the air prior to contacting the electrode surface (red line) and of a pipette at the electrode surface (black line; electrochemical reduction). The suggested current threshold range (dark blue) is between 5 times the maximum noise level and ∼90% of the transport-limited steady-state current (*i*_ss_). The possible region (cyan) is not recommended for the initial SECCM experiments described in this work but can be appropriate for further measurements.

The current threshold, *i*_thresh_, dictates the current value which when exceeded triggers the z-piezo motion to cease. Typically, triggering is based on the absolute value of the current, although this may vary between instruments or be an option in software. Figure 15 shows a schematic of how *i*_thresh_ and *E*_appr_ are chosen, with the suggested *i*_thresh_ value (blue region) chosen greater than the noise level (gray region), but lower than the anticipated current, which is discussed below in detail (black curve shows the steady-state response).

To determine the noise level of the system, we recommend measuring the *i*-*t* response of the pipette suspended in the air before the approach. This is indicated schematically by the red line in Figure 15 from which the maximum noise level, *i*_noise_^max^, is determined. The gray region represents ±5*i*_noise_^max^. Avoid picking *i*_thresh_ values in this range, as it risks a *false trigger* (triggering due to noise before the surface has been reached; see *Running the Computer-Controlled Piezo-Driven Approach* for more details on diagnosing false triggers). NB: While an *i*-*E* measurement is shown in the schematic, an *i-t* curve more closely matches the experimental conditions during the approach at a constant potential.

The current upon meniscus contact with the surface comes from Faradaic and non-Faradaic processes and includes transient and steady-state contributions, thus predicting its precise value is challenging. Nevertheless, the predicted steady-state diffusion-limited current, *i*_ss_, provides an informative benchmark, as given by^52^
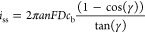
5where *a* is the pipette tip radius, *n* is the number of electrons, *F* is Faraday’s constant (96485 C/mol), γ is the half-angle of the pipette, and *D* and *c*_b_ are the diffusion coefficient and bulk concentration, respectively, of the reactant species. This provides a predictor of the Faradaic current in SECCM experiments with a sufficient overpotential, including for the suggestion for *E*_appr_ given above. For example, for the approximate experimental parameters used in the acquisition of Figure 2, where  is 5 mM, γ is 12°, *a* is 500 nm, *n* is 1, and  is 8.43 × 10^–9^ m^2^/s (ref (75)), *i*_ss_ is ∼−155 pA. The expected transport-limited current can be used to select appropriate amplifier gains (see *Resistor Response* for detailed discussion on amplifier settings).

Figure 15 shows the steady-state voltammogram as a black curve (see eq 11 in ref (52) and the discussion thereof for an analytical description of the full voltammogram). From this figure, we can explore the merits of different choices of *i*_thresh_ and *E*_appr_. As indicated by the regions of “suggested” and “possible” *i*_thresh_/*E*_appr_ values there is no single “correct” setting, but rather a range of reasonable values with differing merits. As discussed above, *i*_thresh_ should be chosen to be greater than the noise level, but less than the anticipated current. As such, in all cases, *i*_thresh_ should be outside the ±5*i*_noise_^max^ range (gray region in Figure 15) to avoid false triggers. Note, offsets in the current will shift the likely false trigger (gray) region vertically, and so should be accounted for in choosing *i*_thresh_. It should now be apparent why such effort was put into the system noise in the *Evaluating the Instrument Electronics* section!

For the initial experiments described in this work, we suggest starting with *E*_appr_ at least 0.1 V beyond *E*^0^ (dark blue region), where the maximal steady-state current (*i*_ss_) occurs. This provides a conservative estimate of the expected transient response on meniscus contact, where additional contributions from non-steady-state mass-transport and non-Faradaic are expected. As the *i*_thresh_ value is increased, the risk of false triggers decreases, but the possibility of a lower than anticipated current causing a tip crash increases. Given that the former results in failing to characterize the sample at one location, while the latter results in the catastrophic end of the experiment, we typically pick conservative values significantly below *i*_ss_. For a system displaying the noise characteristics shown in Figure 5 (+0.1 pA offset, *i*_noise_^max^ = 0.13 pA) and with the predicted *i*_ss_ = −155 pA we would pick *i*_thresh_ as 2 or 3 pA.

More generally, an experiment may require a lower value of *E*_appr_ to avoid undesirable reactions prior to electrochemical characterization, e.g., where the reactions change the electrode surface, such as with electrodeposition,^76^ corrosion,^77^ oxide layer formation/destruction,^78^ or electrode fouling.^79^ Fortunately, for an appropriately denoised setup (see *Open-Circuit Response*) it is typically possible to approach the surface in this potential range due to current contributions beyond those for the steady-state Faradaic process indicated by the black line in Figure 15. Any potential greater than the onset potential will see Faradaic current responses, this response will be larger than the steady-state response for the same potential due to transient development of a concentration profile (see ref (52) for detailed description of transient responses). Even below the onset potential, non-Faradaic currents are typically larger than 5*i*_noise_^max^ and can trigger the termination of the approach (see ref (80) for a detailed discussion of non-Faradaic/charging currents in SECCM). These currents are typically smaller and more challenging to predict compared to those for the redox reaction; hence, our recommendation is to begin experiments in the larger overpotential region. However, picking *i*_thresh_ a small distance above the noise threshold allows experienced experimenters to routinely perform experiments with *i*_thresh_/*E*_appr_ in the “possible” (cyan) region in Figure 15. Picking parameters in this region would be an appropriate follow-on exercise for a reader who has successfully built up confidence in performing the experiments described in this work.

#### Running the Computer-Controlled Piezo-Driven Approach

Once the pipette has been lowered close to the surface and the approach settings are selected, the computer-controlled approach protocol can be run. This piezo-driven motion ends either due to *i*_thresh_ being exceeded or the z piezo reaching its full extension without the current threshold being reached. The latter case indicates that the pipette began the approach insufficiently close to the surface and the pipette is retracted by the piezo. The pipette should be repositioned closer to the surface using the coarse positioners and the computer-controlled approach protocol rerun. Note: it is suggested that the pipette be lowered a distance less than the entire piezo range (e.g., ∼90% full range) to ensure that it will not accidentally contact the surface. Depending on the initial pipette-surface separation and the piezo range, multiple cycles of this inchworm procedure (piezo down → piezo up → coarse positioning down; repeat until trigger) may be needed to place the meniscus in contact with the surface (see Supporting Information section S11 for a schematic of inchworm procedure). On instruments fitted with stepper motors or other electronically controlled actuators, the inchworm procedure is typically automatically implemented, with the cycle repeating without intervention.

**Figure 16:**
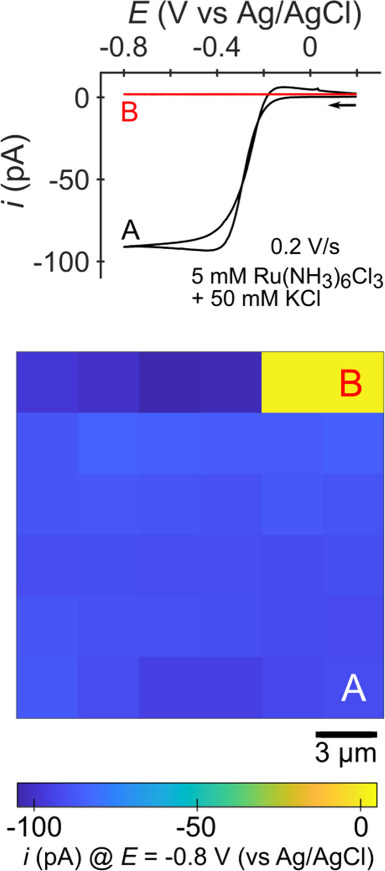
SECCM electrochemical map of the measured current through the HOPG sample, *i*, at *E* = −0.8 V vs Ag/AgCl where two false triggers occurred in the top right corner of the image. The solution in the pipette (pipette radius ≈ 500 nm) was 5 mM Ru(NH_3_)_6_Cl_3_ with 50 mM KCl. The area imaged with SECCM was 18 μm^2^ with 6 pixels for *x* and 6 pixels for *y. i*_thresh_ = 3 pA, *E*_approach_ = −0.8 V vs Ag/AgCl, approach speed = 0.5 μm/s, retraction distance = 2 μm, settling time = 500 ms, and scan rate = 0.2 V/s. Measurements were taken with a Park NX12 (Park Systems, Inc.) instrument using the Park NX12 SICM head internal amplifier (1 kHz bandwidth). Corresponding topography images are shown in the Supporting Information, section S14.

If the approach terminates due to the *i*_thresh_ trigger, the response to an electrochemical measurement indicates whether the surface has been reached or whether a false trigger has occurred. The false trigger response is that of a CV taken with the pipette suspended in the air, as shown in Figure 16, Curve B. In this case, one can repeat the approach protocol, either using the same parameters or with a slightly increased value of *i*_thresh_ to decrease the chance of false triggers. If the meniscus has touched the surface, a nonzero voltametric response corresponding to the redox reaction on the surface is expected. Example CVs of 5 mM Ru(NH_3_)_6_Cl_3_ taken at the surface of HOPG are shown in Figure 2i and Figure 16 Curve A. For this system, desirable CVs should be sigmoidal as those in these figures, but with a limiting current dependent on the pipette size, mediator concentration, and diffusion coefficient as predicted by eq 5.

The half-wave potential and oxidative/reductive nature of the voltammogram will be dependent on the redox mediator chosen. Non-steady-state behavior manifests as hysteresis between the forward and reverse scans and slight peaks in the data^27^ and can be seen in both Figure 2 and Figure 16. While this is of no concern for these introductory measurements, it could be diminished by using a slower scan rate (see ref (27) for a discussion on scan rate dependence). Pay careful attention to the current magnitude, if it is much larger than expected (>5× than predicted), this suggests a tip crash, i.e., the glass pipette contacted the electrode surface and sustained damage (smaller differences might be due to inaccuracies in pipette tip characterization or evaporation leading to enhanced concentrations). See Supporting Information, Section S12 for examples of undesirable voltametric responses due to broken tips and/or droplet spreading. Tip damage can be assessed with optical microscopy, or through a resistance measurement (see *Measuring the i-t and i-E Response of a Nanopipette in Bulk Solution*); NB: the latter will leave the pipette covered in electrolyte and unsuitable for further SECCM experiments. If the tip is broken, it is unusable for imaging, and a new one should be pulled and filled for subsequent experiments. If repeated tip crashes occur, it is suggested to use more conservative approach parameters (see *Settings for the Computer-Controlled Piezo-Driven Approach*).

As stated above, it is our experience that beginning SECCM users frequently position the pipette a significant distance from the surface (often 5–10 times the piezo range) necessitating multiple repeats of the inchworm cycle leading to extended approach durations. We encourage beginning experimenters to determine the total distance moved during all portions of the approach. This distance should be related to the optically observed initial tip–surface separation after manually lowering (see *Lowering the Pipette toward the Surface)*. By relating these distances, a beginning experimenter will rapidly gain the necessary experience to position the pipette at a suitable distance from the sample that neither risks unintentional pipette-surface contact nor unnecessarily long approaches (our observations show this experience comes in <10 experiments).

### Imaging the Surface

The process of imaging the sample surface via SECCM is shown schematically in Figure 1 and is outlined in the flowchart shown in Figure 13, with details described in the following subsections.

#### Imaging Parameters

The imaging parameters chosen for an SECCM experiment determine the area mapped, the experimental duration, and the success of the experiment. While the exact names of the parameters will vary from instrument to instrument, the major settings determine the dimensions and number of *x* and *y* pixels in the image, approach settings (these are as described in *Settings for the Computer-Controlled Piezo-Driven Approach*), settling time (quiet time) before the electrochemical measurement, and retraction distance. Appropriate choices of the parameters will depend on the experimental system being measured, with appropriate considerations discussed below. One should also set up the electrochemical technique to be performed when the meniscus is in contact with the electrode surface; however, given the range of possibilities for this, their discussion is beyond the scope of this work. Herein, we choose voltammetry over an appropriate potential window at an appropriate scan rate to give (close to) steady-state behavior. Minor settings, e.g., *xy* speed, vary from instrument to instrument, these exert less influence on the experimental outcome – to learn their function we refer readers to their instrument’s documentation.

The array of *xy* points where electrochemical measurements will be performed is determined by the *xy* dimensions of the SECCM image, the offset (typically given as the location of the image center or of a corner), and the number of pixels in each direction. The approach settings are the same as those used in the approach protocol (see *Settings for the Computer-Controlled Piezo-Driven Approach*) and we recommend beginning with successful settings determined in this section. The settling time is the amount of time between the *z* movement stopping from the current threshold being reached and before the electrochemical measurement is taken (see Figure 1 bottom), this allows the meniscus to stabilize (a conservative settling time of 500 ms is suggested in this work). The retraction distance is the *z* distance the pipette retracts after completion of the electrochemical measurement and before moving to the next *xy* coordinate. The retraction distance must be sufficient for the meniscus to detach from the surface to avoid complications that may end up in pipette tip damage. Longer retraction distances will lead to longer approaches to subsequent locations, increasing the overall duration of imaging. We recommend starting with a moderately conservative retraction distance of ∼5× the pipette radius.

We recommend first-time SECCM experimentalists start with a small image (e.g., ∼5 μm^2^ area, 5 × 5 pixels) and image nonoverlapping points. Residue on the surface from previous approaches can lead to instability in the sample wetting, complicating interpretation and performance, and so is best avoided. For this reason, we also suggest performing the image away from the area addressed in *Approaching the Surface*. The scan duration scales with the number of pixels and so a 10 × 10 image will take 4 times longer than a 5 × 5 image (same settings). For this reason, when extending to larger scans it is good practice to estimate the acquisition time by considering the time to measure one pixel and multiply by the number of pixels in the image. Estimates can come from the duration of a previous experiment (Time for one pixel = Total imaging duration/no. pixels) or analytically (Time for one pixel = Time for approach + Time for electrochemical measurement + Time for retraction + Settling time, where Time for Approach ≈ Retract Distance/Approach Speed and Time for retraction ≈ Retract Distance/Retract Speed).

#### Results of Imaging Experiments

Once the imaging parameters have been set, one can run the imaging protocol and examine the resultant data. Common problems encountered when imaging, which are discussed below, include false triggers, the meniscus failing to detach from the surface between points, and tip crashes. If the pipette tip crashes during the experiment, one should adjust the experimental settings before the next experiment to avoid the same mode of failure. It may take a handful of iterations to converge on stable imaging parameters for a new system.

The result of a successful SECCM voltammetric image was shown in Figure 2. The top portion shows examples of CVs taken at three of the points (as labeled). For the 100–500 nm radius pipettes recommended in this work, slight peaks in the voltammogram can be observed when high scan rates are used as discussed in *Running the Computer-Controlled Piezo-Driven Approach*. These peaks can be mitigated by decreasing the scan rate. We find scan rates ≤ 1 V/s give steady-state (no peaks) for 100–500 nm radius pipettes and faster scan rates can be used for smaller pipettes. The middle image of Figure 2 represents the current at the switching potential (*E*_λ_ = −0.8 V vs Ag/AgCl) measured at each location. Note that while the pixels in the image are shown as tessellated squares, this does not accurately reflect that the characterization took place in the approximately circular area wetted by the meniscus. As such, plotting each pixel as a circle with an area equal to that of the area wetted by the meniscus is another common alternative plot, an example of which is shown in Figure S14.

The lower portion of Figure 2 shows the topographic data (height at which the meniscus contacted the sample), from which we observe that the upper portion of the image is ∼3 μm higher than the lower portion, but there is relatively little other topographic information visible. We note that this height difference may be due to the sample not being mounted orthogonally, or through (thermal) drift. A small tilt in the sample has little impact on the wetted area or overall form of the approach curve. However, a sample tilted toward/away from the tip will cause a slightly higher demand on the piezo range as the length of an approach will be slightly shorter/longer than the distance the tip was retracted at a previous location on the surface. Such tilt is unlikely to cause issues unless taken to extremes (a 100 μm line with a 2° tilt would have a 3.5 μm height difference (=100 μm × tan(2°)). A tilt in the resulting topographic image can make it harder to see small topographic features. Ideally, a sample is mounted perfectly flat; however, subtracting a plane (or similar) from the topographic data in postprocessing, as is commonplace with other SPMs such as AFM,^63^ will aid in visualizing small topographic features.

Figure 16 shows an example of an SECCM image that was a qualified success. While reproducible measurements were acquired at most points, false triggers occurred at the point labeled B and the one to its immediate left. The electrochemical measurement taken at the end of the approach is used to determine whether the meniscus contacted the surface or not. The voltammogram from point A shows the response when the meniscus reached the surface, with a well-defined wave at the appropriate potential. Curve B shows the flatline response of a pipette suspended in air, corresponding to a point where a false approach/trigger occurred. While occasional false triggers may be tolerable in some circumstances (they do not result in tip damage); however, they may be avoided with a higher *i*_thresh_.

**Figure 17:**
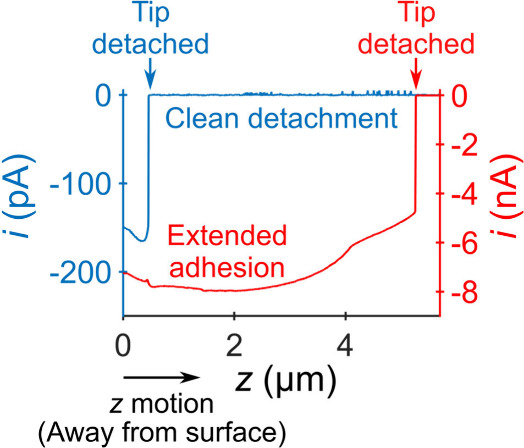
*i*-*z* response of a ∼500 nm radius pipette filled with 5 mM Ru(NH_3_)_6_Cl_3_ with 50 mM KCl being moved away from the surface for a clean detachment (blue) and extended adhesion (red). The left *y*-axis corresponds to *i* (pA) measured for the clean detachment, while the right *y*-axis corresponds to *i* (nA) measured for the extended adhesion. The tip is in contact with the electrode at *z* = 0 μm. Electrode set at *E* = −0.8 V vs Ag/AgCl for all *z*. Retraction speed is ∼4.5 μm/s. Measurements were taken with a Park NX12 (Park Systems, Inc.) instrument using the Park NX12 SICM head internal amplifier (1 kHz bandwidth).

For stable, reproducible SECCM imaging, it is important that the meniscus detaches from the surface between points. The experimental *i* vs *z* data obtained during the retraction phase, such as is shown in Figure 17, can give information on whether the pipette is detaching as desired. The blue curve shows a clean detachment where the pipette detaches from the surface (as indicated by the return to zero current) in ∼0.5 μm. Note that the potential of the electrode is held at a reducing potential (*E* = −0.8 V) so that the return to zero current can be seen; at potentials where the redox reaction does not occur on the electrode, the detachment is often not clearly visible. While the exact details of the detachment are not fully reported in the literature, they likely depend on multiple parameters, such as the pipette radius and the hydrophobicity of the surface. We find that for the experiments described herein, detachment is observed within ∼1.5–5 pipette radii. Note, while a retract distance of ∼750 nm would be adequate for the ∼500 nm radius pipette used to acquire the data Figure 17 (clean detachment) to have detached from the surface, we suggest beginning with more conservative (longer) retract distance.

The red curve in Figure 17 shows an extended adhesion from a deliberately damaged tip, taking ∼5 μm for the meniscus to detach (note the different current scales). An extended adhesion is often due to droplet spreading, which can be caused by tip breakage, a hydrophilic sample, or low humidity levels. Humidity levels >60% are ideal to lower evaporation rates and the formation or electrolyte crystals.^81^ If one encounters humidity issues, we recommend controlling humidity by using a moat of water surrounding the sample (see Figure S1 for example), using a custom-made environmental chamber such as that described by Jayamaha et al.^9^ (and shown in Figure S2) or using oil-immersion SECCM.^77^ A sudden change in the distance at which the meniscus detaches suggests instability in the meniscus and is often accompanied by unstable voltammetry and/or larger currents, which are all suggestions that the pipette tip has become damaged by contacting the surface. As shown in Figure 17 the current measured while in contact with the surface for the deliberately broken (extended adhesion) tip compared to the desired (clean detachment) tip are much higher (∼8 nA compared to ∼150 pA), which is an indicator of a broken tip.

#### Optimization of Imaging Parameters

The parameters described above were chosen conservatively to give the best chance of success on these first images. Once successful images can be obtained routinely, the experimenter is encouraged to consider the trade-offs described above to improve upon and optimize these parameters to minimize false triggers and shorten experimental durations, while maintaining stability. When looking at optimization, consider the overall duration of each portion of the imaging protocol when looking to optimize. For example, if 95% of the time is spent acquiring multiple slow-scan voltammograms, there is little to be gained by approaching at a higher speed (and increased likelihood of a tip crash), whereas sweeping the voltage more rapidly, decreasing the number of voltammetric cycles, or decreasing the number of pixels in the image, will shorten the experiment considerably and may be appropriate if similar conclusions can be obtained.

### Common Artifacts in SECCM Responses

As discussed throughout this text, there are many common artifacts in SECCM measurements that arise from various origins. Table 1 compiles these artifacts and provides possible causes and sections/figures in the main text where they are discussed. Note that the *Unstable current* artifact may be observed between locations as large erratic variation in *i*_ss_ (e.g., > 50%) (NB: this is only applicable when using an outer-sphere redox mediator and a uniform sample, such as the example system considered in this work) or within a single voltammogram (rapid changes in current during a single voltammogram).

**Table 1:** Common Artifacts Seen in SECCM Responses, Their Possible Cause(s) and Remedies, and the Location They Are Discussed in the Text

Artifact	Possible cause(s)	Remedy	Location of relevant discussion
Flatline response after approach (e.g., Figure 16 curve B)	• Bad electrical connections	• Address bad connections	• *Evaluating the Instrument Electronics*
	• False trigger	• Increase *i*_thresh_	• refs (32) and (33)
		• Denoise	• *Settings for the Computer-Controlled Piezo-Driven Approach*
			• *Results of Imaging Experiments*
Current (*i*_ss_) > 5× predicted (e.g., Figure S13)	• Pipette damage	• Prepare fresh pipette	• *Running the Computer-Controlled Piezo-Driven Approach*
	• Droplet spreading	• Alter approach settings	• *Results of Imaging Experiments*
		• Control humidity	
Unstable current (e.g., Figure S13)	• Droplet spreading	• Control humidity	• *Results of Imaging Experiments*
	• Low humidity	• Pipette silanization	• *Filling Pipettes*
	• Crystal formation on tip	• Increase electrolyte surface tension^82^	
Non-steady-state voltammetry	• Pipette damage	• Prepare fresh pipette	• *Results of Imaging Experiments*
	• Droplet spreading (meniscus footprint radius ≫ pipette radius)	• Control humidity	• refs (27) and (82)
	• Planar mass transport > radial mass transport^27,52^	• Decrease scan rate	
		• Pipette silanization	
		• Increase electrolyte surface tension^82^	

### Data Processing and Visualization

Once the imaging data has been collected, one can begin data processing and visualization using one of a multitude of different software and packages. The multiple quantities being measured at any time during a SECCM image (*xyz* position, *E*, *i*, and *t*) can be plotted either in the imaging software, third-party software, or by self-written scripts. Given the diversity of output formats and possibilities, we do not go into detail here but suggest those struggling to visualize data consult the documentation of their instrument, contact its supplier, or consult a user of the same instrument.

## Conclusion

A detailed step-by-step guide to SECCM imaging of a well-behaved model system was given. This introduction to SECCM experimentation provided a self-contained foundation suitable for those without prior SECCM experience, including those who lack support from experienced individuals. A systematic presentation gives detailed descriptions of each experimental step, example data (desired results and commonly encountered problems), and troubleshooting strategies. SECCM experiments were divided into three major portions (electronic instrumentation, preparation of pipettes, and approaching the surface and imaging), each presented through step-by-step flowcharts outlining individual steps and troubleshooting pathways. Key literature references were provided, directing the reader to a detailed discussion of important topics. Pipette filling and positioning the pipette close to the sample (coarse positioning) were highlighted as typical pain points for beginner experimenters, with strategies for rapidly overcoming them provided.

While this work describes procedures for single-barrel SECCM experiments, adaption to dual-barrel SECCM,^28^ which allows for imaging samples containing mixtures of conducting and insulating regions, would require only minor modifications. Readers of this work gain an understanding of the operating principles of SECCM and of experimental details. Those successfully following along will gain confidence and competence in acquiring SECCM images, learn to troubleshoot experimental problems, and understand how appropriate experimental parameters can be chosen. Following successful completion of the steps in this work, the experimenter interested in applying their newfound expertise to a broader range of experimental systems is encouraged to consult *Practical Guidelines for the Use of Scanning Electrochemical Cell Microscopy (SECCM)*.^9^ We hope to provide an accessible route for a broad range of researchers to adopt these powerful electrochemical tools.

## Data Availability

Data for this article are available at Dryad at DOI: 10.5061/dryad.3j9kd51v4

## Supporting Information Available

S1 – pathways for using this work, S2 – experimental details, S3 – example instrument setups, S4 – open-circuit *i-t* responses using different current amplifiers, S5 – open-circuit *i-E* responses at different scan rates, S6 – resistor response experiment setup, S7 – zoomed-in voltammetric response of a resistor, S8 – pipet optical microscopy, S9 – zoomed-in views of experimental setup, S10 – using cameras to aid pipet approach, S11 – *z* movement during an inchworm approach, S12 – undesirable CV responses, S13 – SECCM electrochemical map plotted as isolated circles, S14 – *z*-height map from an image containing false-trigger events, S15 – printable flowcharts, S16 – description of supporting videos, S17 – Vocabulary (PDF)
Gently brushing the threads of a screw against a nanopipette to release bubbles (MP4)
Agitating a nanopipette with serrated tweezers to release the bubbles (MP4)
Backloading method of filling a pipet described in *Filling pipettes* and shown schematically in Figure 7 (MP4)
