# Supplementary material for: A Tutorial for Scanning Electrochemical Cell Microscopy (SECCM) Measurements: Step-by-Step Instructions, Visual Resources, and Guidance for First Experiments
Source: ACS Meas Sci Au. 2025 Mar 27;5(2):160–77. doi: 10.1021/acsmeasuresciau.4c00091 (PMC12006954; doi:10.1021/acsmeasuresciau.4c00091)
Supplement: Supplementary file 1 — tg4c00091_si_001.pdf [file tg4c00091_si_001.pdf]

Supporting Information for:

A Tutorial for Scanning Electrochemical Cell Microscopy (SECCM) Measurements: Step-by-Step Instructions, Visual Resources, and Guidance for First Experiments

Kamsy Lerae Anderson and Martin Andrew Edwards*

Department of Chemistry and Biochemistry, University of Arkansas, Fayetteville, AR 72701, USA

*maedw@uark.edu

Table of Contents

S1: Pathways for Using this Work S2
S2: Experimental Details S3
S3: Example Instrument Setups S6
S4: Open-circuit *i-t* Responses Using Different Current Amplifiers S9
S5: Open-circuit *i-E* Responses at Different Scan Rates S10
S6: Resistor Response Experiment Setup S11
S7: Zoomed-in Voltammetric Response of a Resistor S12
S8: Pipette Optical Microscopy S13
S9: Zoomed-in Views of Experimental Setup S14
S10: Using Cameras to Aid Pipette Approach S15
S11: *z* Movement During an Inchworm Approach S17
S12: Undesirable CV Responses S18
S13: SECCM Electrochemical Map Plotted as Isolated Circles S19
S14: z-height Map from an Image Containing False-trigger Events S20
S15: Printable Flowcharts S22
S16: Supporting Videos S25
S17: Vocabulary S26
References S27

S1: Pathways for Using this Work

As discussed in the introduction of the main text, the steps presented in this work are described in chronological order and are aimed at individuals who are new to SECCM experiments and with an instrument they either do not know or trust. However, for those who are not in this situation, it may be possible to skip some sections. Below we describe alternate pathways for using this work depending on the experience and state of the instrument that the experimenter has access to.

S1.1 Alternate Pathway 1

If one has a fully functioning instrument, knows how to prepare quasi-reference counter electrodes, and is confident in preparing and mounting pipettes, we recommend skipping to the *SECCM Experiment* section and following the steps described in the Figure 13 flowchart.

S1.2 Alternate Pathway 2

If one has a fully functioning instrument and is confident in preparing electrodes but lacks experience in preparing quasi-reference counter electrodes and/or mounting pipettes, we recommend first reading the sections *Quasi-reference Counter Electrodes (QRCEs)* and *Mounting the Pipette*. Then skipping to the *SECCM Experiment* section and follow the flowchart shown in Figure 13.

S1.3 Alternate Pathway 3

If one has a fully functioning instrument but has never prepared pipettes, we recommend starting at the *Pipettes* section and following the flowcharts shown in Figure 7 and then Figure 13 to the end.

S2: Experimental Details

Details of the experimental setups used to obtain data shown in the main text figures are provided below. A cumulative list of all materials used in all experiments and their suppliers can be found in S2.1 List of Experimental Materials.

SECCM imaging (Figures 2 and 16)

Instrument: Park NX12 (Park Systems, Inc.)

Amplifier used: Park NX12 SICM head internal amplifier (1 kHz bandwidth)

Gain: 10^9 V/A

Scan rate: 0.2 V/s

Pipette tip radius: ~500 nm

Sample: freshly-cleaved HOPG (see reference 1 for additional details on sample cleaving)

QRCE: Ag/AgCl

Solution: 5 mM $Ru(NH3)_6Cl_3$ with 50 mM KCl

Pipette pulling: Sutter P-2000 laser puller; parameters: HEAT = 700, FIL = 4, VEL = 60, DEL = 145, PUL = 150

The sample was mounted inside a small petri dish and towels moistened with water were used to surround the sample to aid in humidity control (See Figure S1 for an image of experiment setup).

Open-circuit measurements (Figure 5)

Instrument: Park NX12 (Park Systems, inc.)

Amplifier used: Park NX12 SICM head internal amplifier (1 kHz bandwidth)

Gain: 10^9 V/A

Voltammetric characterization of a 100 MΩ resistor (Figure 6)

Instrument: Home-built SECCM instrument

Resistor: 100 MΩ thick film through-hole resistor (Mouser Electronics), tolerance 1%

Amplifier used: DDPCA-300 (FEMTO Messtechnik GmbH, Germany)

Bandwidth: 150 Hz (full BW at chosen gains)

Gain: A, B, and E) 10^8 V/A C) 10^9 V/A

Scan rate: 0.2 V/s

Digitized response (Figure 6D) was calculated assuming 2 V resolution.

Experimental Details for Electrical Characterization of a Pipette in Bulk Solution (Figures 11 and 12)

Instrument: Park NX12 (Park Systems, Inc.)

Amplifier used: DDPCA-300 (FEMTO Messtechnik GmbH, Germany)

Gain: 10^7 V/A

Bandwidth: 400 Hz (full BW)

Scan rate: 0.1 V/s

Pipette tip radius: ~500 nm

QRCE and bath electrode: Ag/AgCl wires

Solution: 0.1 M KCl

Pipette pulling: Sutter P-2000 laser puller; parameters: HEAT = 700, FIL = 4, VEL = 60, DEL = 145, PUL = 150

S2.1 List of Experimental Materials

Below is a comprehensive list of the materials used to perform all the experiments described within this work along with the suppliers used.

HOPG (SPI Supplies) Grade SPI-3 10×10×1 mm thick

Metal specimen discs, 20 mm diameter (Ted Pella)

Conductive liquid silver paint (Ted Pella)

Copper foil tape, 1/2-inch x 33 FT (AIYUNNI)

Double-sided tape (Amazon Basics)

Transparent tape (BestSource OfficeSupplies)

Test lead set and alligator clips (WGGE)

BNC coaxial cables of various lengths (50 cm - 2 m), Impedance 50 Ohms (Pomona Electronics)

1 mL luer slip tip syringe (BH Supplies)

MicroFil flexible needle (WPI, 28 gauge, MF28G67)

Hexaamineruthenium(III) chloride, 99% (Strem)

Potassium chloride (EMD Millipore)

18MΩ·cm Millipore water obtained from a Direct-Q 3UV water filtration system

Silver wire, diam. 0.25 mm, ≥99.99% trace metals basis (Sigma Aldrich)

Bleach (5-9% sodium hypochlorite; Clorox)

P-2000 Laser puller (Sutter Instrument)

Quartz glass capillary O.D. 1.00 mm I.D. 0.70 mm 7.5 cm length (Sutter Instrument)

100 MΩ thick film through-hole resistor (Mouser Electronics) Tolerance 1%

Optical microscope 40X-1000X Magnification (AmScope, M150C-PS25)

Scanning electrochemical cell microscope including current amplifier (e.g., Park Systems NX12)

Digital multimeter (VC830L)

We recommend this as a *starter kit* for performing SECCM experiments. Substitutions of suppliers and varying the choice of redox species (see *Suggested System for First SECCM Measurements* for requirements) are encouraged.

S3: Example Instrument Setups

Figure S1 shows the experiment setup used in acquiring the data shown in Figure 2 and Figure 16 of the main text. In this setup, the HOPG sample is mounted on a metal disk which was electrically connected to a Park Systems NX12 microscope using copper tape, wires (red and white), and alligator clips. Note, a shorter connecting wire would be ideal to minimize noise pickup and stray capacitance.

A moat-and-pedestal configuration was used to provide a humid environment around the pipette tip/sample. The metal disk was mounted onto a small plastic box (pedestal) (Ted Pella) using double-sided tape, which was attached inside a small petri dish using double-sided tape. Kimwipes (Kimtech Science) were moistened with water and inserted in the petri dish around the sample. This setup also employs a top-view camera (as labelled).

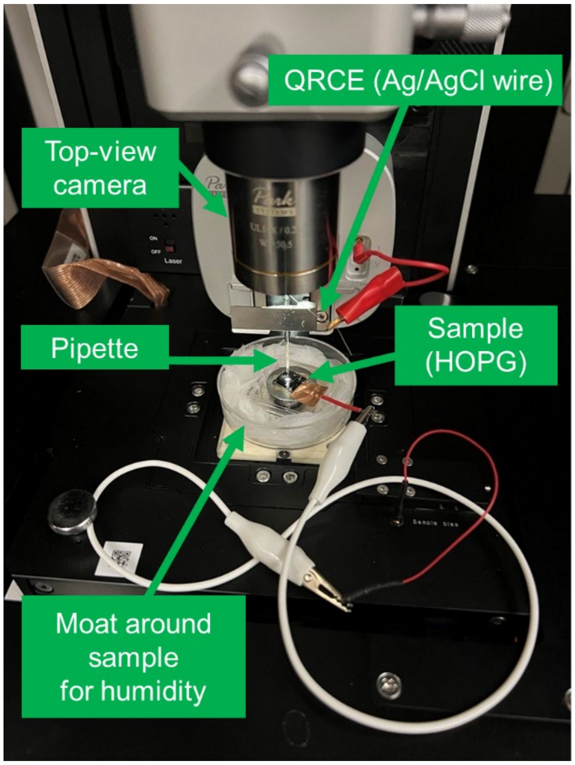

Figure S1. Park Systems NX12 microscope SECCM setup with moat around sample for humidity control. This setup was used in acquiring the data shown in Figures 2 and 16 of the main text.

Figure S2 shows an example home-built SECCM instrument setup including a custom-built environmental chamber as discussed in *Results of Imaging Experiments* of the main text (image courtesy of Prof. Cameron Bentley, Monash University). A side-view camera is included within this experimental set up (as labelled).

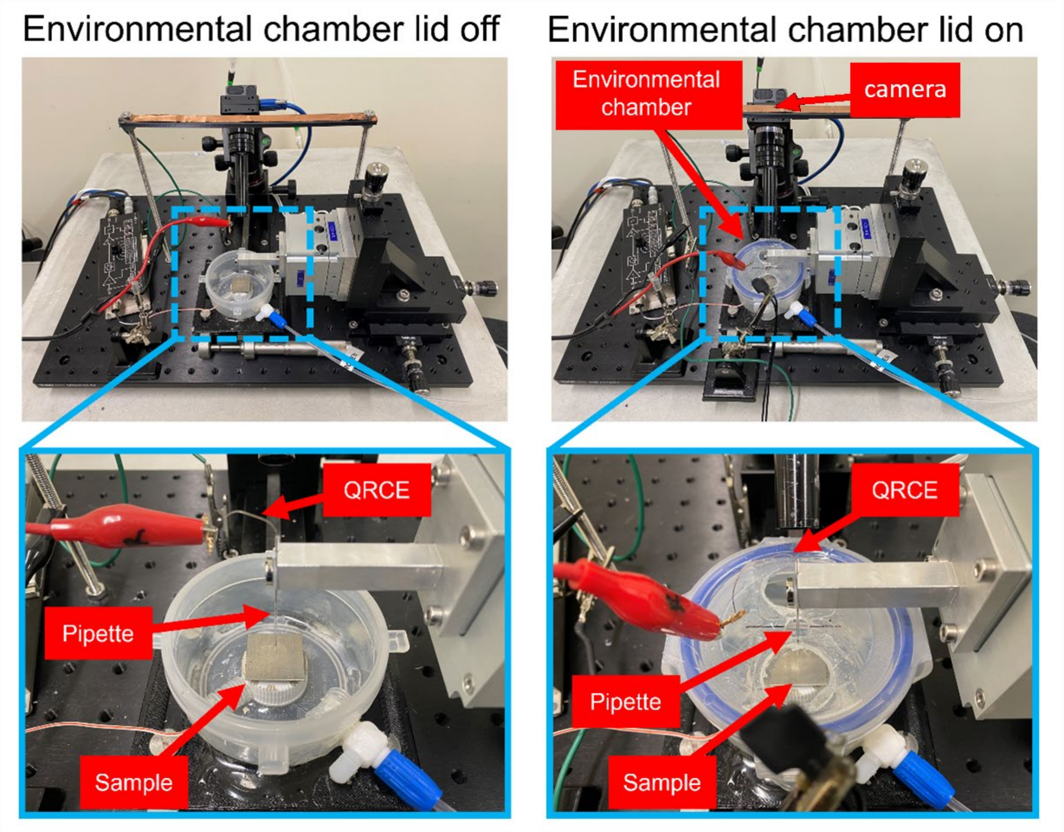

Figure S2. Home-built SECCM instrument setup containing a custom-built environmental chamber (discussed in *Results of Imaging Experiments,* See References 2 and 3 for more details) with the lid off (left) and on (right). The bottom pictures show a zoomed-in view of the setup with arrows labelling the pipette, sample, and QRCE. Thanks to Prof. Cameron Bentley (Monash University) for the images.

Figure S3 shows an example home-built SECCM instrument setup with a 3D-printed sample holder that has a moat for holding liquid to help with humidity control in experiments, as discussed in *Results of Imaging Experiments* of the main text. A side-view video microscope/camera is included in this experimental setup.

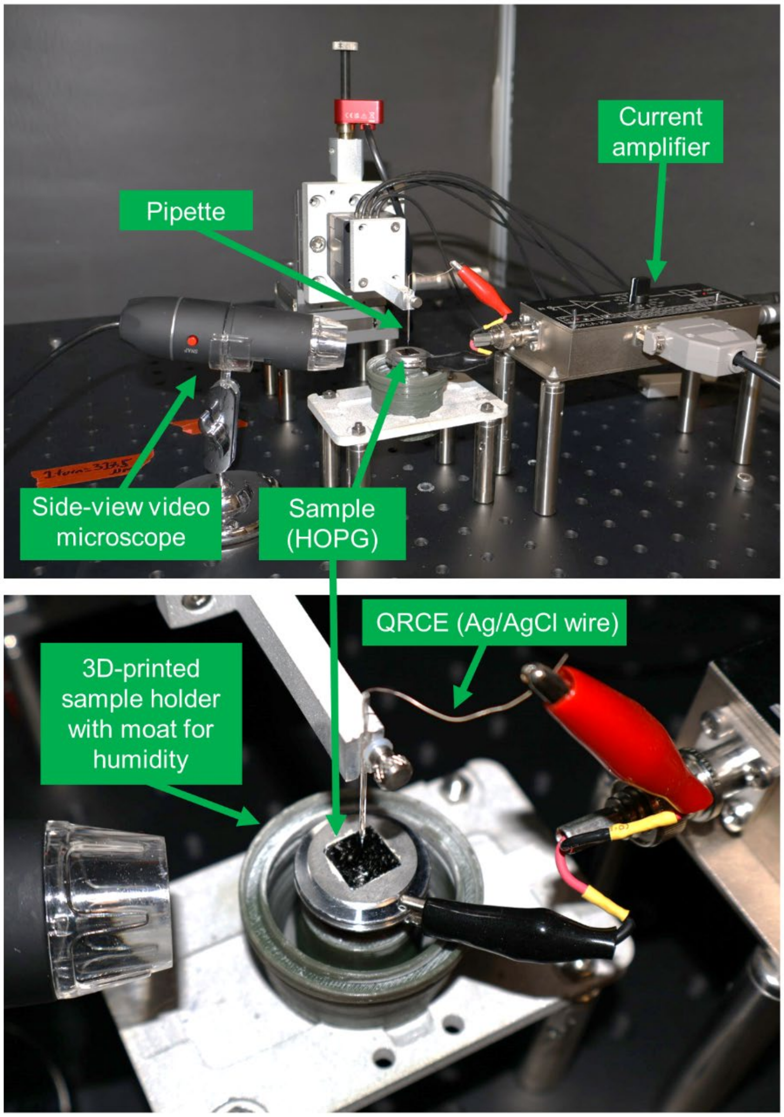

Figure S3. Home-built SECCM instrument setup containing a 3D-printed sample holder that has a moat for holding water to aid in humidity control (discussed in section *Results of Imaging Experiments* in the main text, see References 2 and 3 for more details). The bottom picture shows a zoomed-in view of the setup.

S4: Open-circuit *i-t* Responses Using Different Current Amplifiers

Figure S4 shows the open-circuit *i-t* responses of three different current amplifiers: Park NX12 SICM head internal amplifier (Park Systems, inc.), DDPCA-300 (FEMTO Messtechnik GmbH, Germany), and DLPCA-200 (FEMTO Messtechnik GmbH, Germany). The blue curve corresponds to the desired curve shown in Figure 5A of the main text. Low noise levels are achieved with each amplifier: 0.091 pA RMS for the Park NX12 internal amplifier, 0.088 pA RMS for the DDPCA-300 amplifier, and 0.239 pA RMS for the DLPCA-200 amplifier. All current amplifiers show a small offset of <0.5 pA adequate for the SECCM measurements described in the main text (See *Evaluating the Instrument Electronics*).

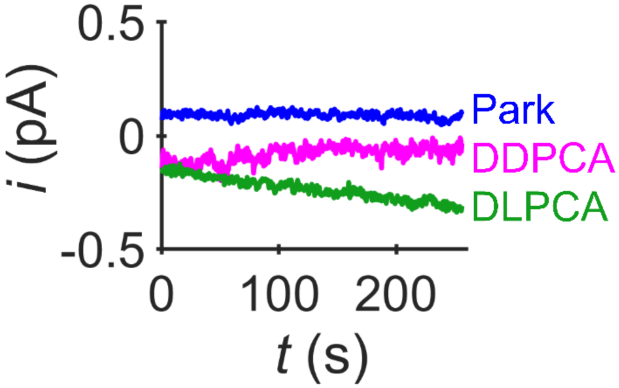

Figure S4. Open circuit *i-t* responses (E = 0 V) from 3 different amplifiers. Park NX12 SICM head internal amplifier (Park Systems, inc.) (blue), DDPCA-300 (FEMTO Messtechnik GmbH, Germany) (magenta), and DLPCA-200 (FEMTO Messtechnik GmbH, Germany) (green). The Park NX12 SICM head internal amplifier operates with a 1 kHz bandwidth, and full bandwidth (150 Hz for DDPCA-300 and 1.1 kHz for DLPCA-200) was used for both FEMTO amplifier measurements. The gain setting for all amplifiers was 10^9 V/A.

S5: Open-circuit *i*-*E* Responses at Different Scan Rates

The blue points in Figure S5 show the capacitive current measured from voltammetry at open circuit vs scan rate. Voltammetric data for the lowest three scan rates are shown in Figure 5B of the main text (10 V/s voltammogram not shown). The red line is the least-squares best fit to the experimental data that passes through the origin. From the slope of the line (0.07 pA/(V/s)) and simplification of the units we determine the stray capacitance as 0.07 pF.

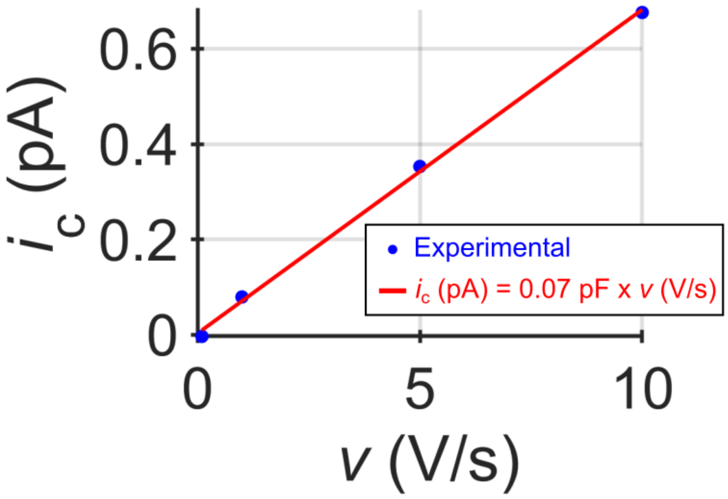

Figure S5. Capacitive currents measured at different scan rates in an open circuit configuration. Voltammetric data from Figure 5B in the main text (10 V/s voltammogram not shown). Details of the measurements are provided in S2: *Experimental Details* and *Open-circuit Response* in the main text.

S6: Resistor Response Experiment Setup

Figure S5 shows two example experiment setups for measuring the response of a resistor, as discussed in the *Resistor Response* section of the main text. Details on the 100 MΩ thick film through-hole resistor shown in the LHS image are available in *S2.1 List of Experimental Materials.* The resistor shown in the RHS is a 100 MΩ C-AFM kit resistor (Park Instruments). The data shown in Figure 6 of the main text were collected using the home-built instrument and 100 MΩ thick film through-hold resistor as shown in Figure S2 left (see *S2: Experimental Details: Voltammetric characterization of a 100 MΩ resistor*, for full experimental details).

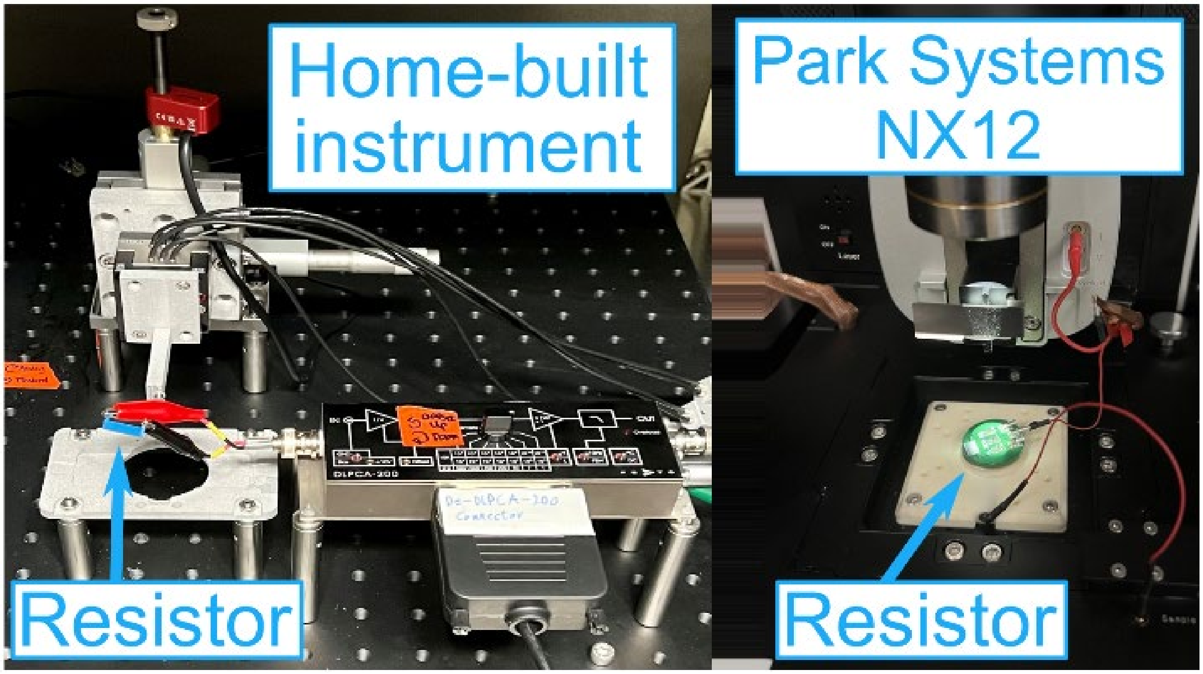

Figure S6. Two different 100 MΩ resistors connected into home-built (left) and commercial (Park Systems NX12) (right) SECCM instruments. Red and black alligator clips are used to connect the resistor in the home-built instrument.

S7: Zoomed-in Voltammetric Response of a Resistor

Figure S7 shows a zoomed-in view of the *desired response* of a 100 MΩ resistor, which was shown in Figure 6 of the main text. With this view, a slight (~0.6%) deviation between the slope of the experimental (red solid) and response to Equation 1 can be seen (black dashed), which is consistent with the 1% tolerance of the resistor used. The experimental data consist of the linear response of an ohmic conductor superimposed with a 'rectangular' current-voltage response centered vertically around zero current that is expected of a capacitor (see reference 4 section 1.6 for details of the capacitor response). We attribute the capacitance contribution to the current responses to stray capacitances, which were also observed at open circuit (see Figure 5B in the main text), offering a parallel current path.

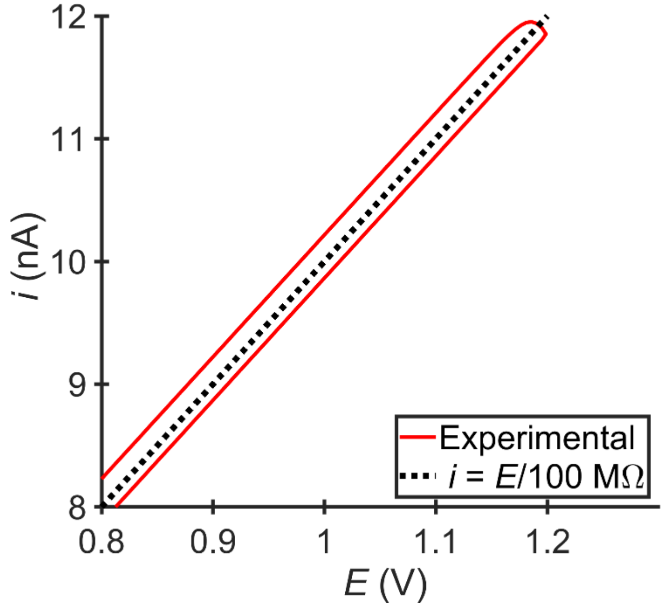

Figure S7. Zoomed-in view of the experimentally measured 'desired' current-voltage response of a 100 MΩ resistor (red line) compared to the response calculated by Ohm's law (black dotted line). These data represent a zoom-in of those shown in Figure 6 of the main text.

S8: Pipette Optical Microscopy

Figure S8 shows example optical micrographs of 3 nanopipettes that are broken very close to the tip. These breaks are less obvious than the broken pipette shown in Figure 8 of the main text, but the jagged, blunt tip that can be observed in the optical microscope indicates that the tip has a radius greater than the expected radius (500 nm). This indicates that the tip is broken, and a new pipette should be pulled and filled for further experiments (compared to the completely filled unbroken shown in the top left of Figure 9 in the main text).

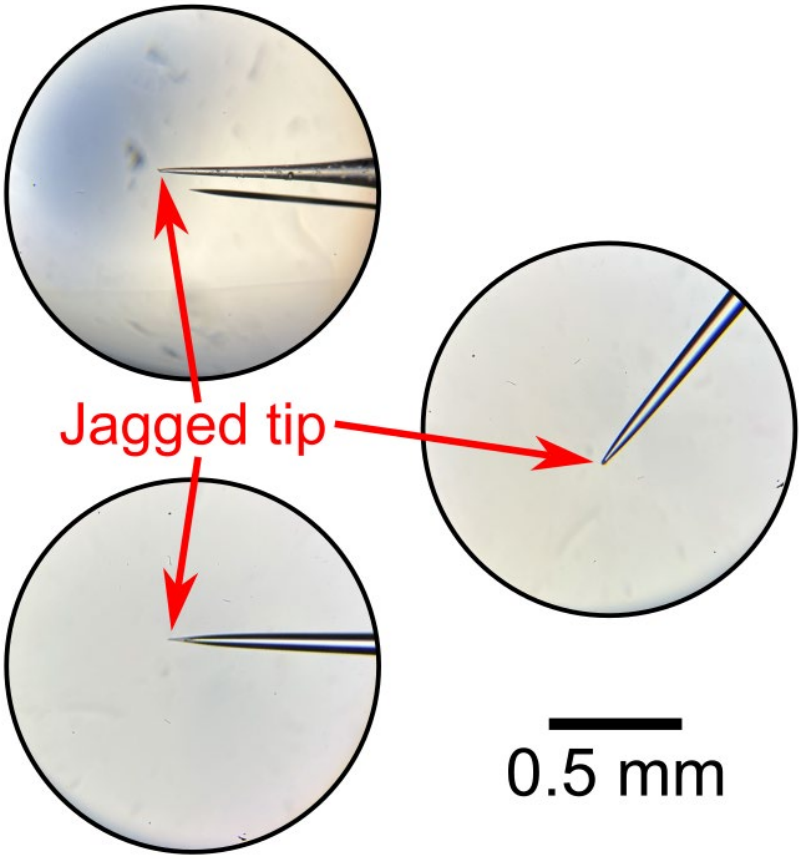

Figure S8. Optical micrographs of pipette which are broken at the very tip. ~500 nm radius pipettes prior to damage. Black needle in top left micrograph is the eyepiece pointer. Magnification factor of objective lens is 10× and magnification factor of the eyepiece lens is 10×.

S9: Zoomed-in Views of Experimental Setup

Figure S9 shows zoomed-in views of Figure 14 of the main text, which allow additional details to be more easily observed. The left portion shows a nanopipette mounted into a Park Systems NX12 microscope with an Ag/AgCl wire QRCE inserted and electrically connected (discussed in *Quasi-reference Counter Electrodes (QRCE)* of main text) to the microscope with an alligator clip and red wire. The right portion shows a HOPG sample which is electrically connected to complete the circuit. See the *Sample Preparation* section of the main text for more details on how the sample was mounted.

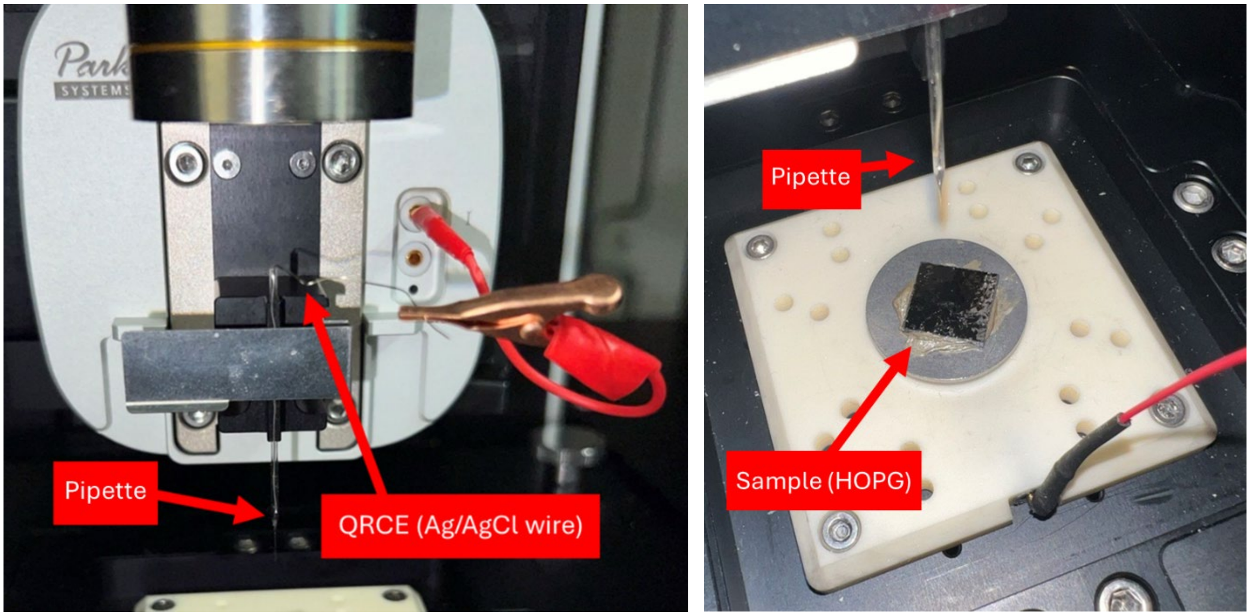

Figure S9. Zoomed in views of the experimental setup shown in Figure 14 of the main text. (**Left**) Ag/AgCl wire inserted into a pipette and electrically connected in a commercial SECCM instrument (Park Systems NX12). **(Right)** HOPG sample affixed to a steel disk using Ag epoxy (See *S2.1 List of Experimental Materials* for supplier details). The disk is magnetically attached to the sample holder (white square). A hidden internal electrical connection goes from the magnetic mount to the socket on the bottom edge allowing the sample to be connected via the pin attached to the red wire.

S10: Using Cameras to Aid Pipette Approach

Figure S10 shows two images of a nanopipette captured by a side-on video microscope. The images are taken before (left) and after (right) a 25 µm movement of the pipette towards the sample. The difference in the pipette position between the two images provides a ruler by which distances in the images can be measured. We find making such comparisons effective in delivering an appreciation for distances visualized by a specific optical configuration.

In this example, the reflection of a nanopipette in the HOPG sample is clearly visible and is labelled in the left-hand image. While not all samples are reflective, a side-on camera can still provide some useful information about the pipette sample separation. When it is visible, the separation between the tip and its reflection can be used to provide a straightforward estimate of the pipette-surface separation. In this case, moving the tip down 25 µm approximately halved the separation, suggesting that the tip has moved from ~50 µm to ~25 µm from the surface. Note, however, that as it is not possible to resolve the nanometric end of the pipette the true separation may be somewhat closer. A conservative approach should be employed when positioning the pipette to avoid damaging it. Leaving a tip-sample separation of at least 10 µm during the coarse approach stage is suggested as little benefit is gained from attempting to position them closer.

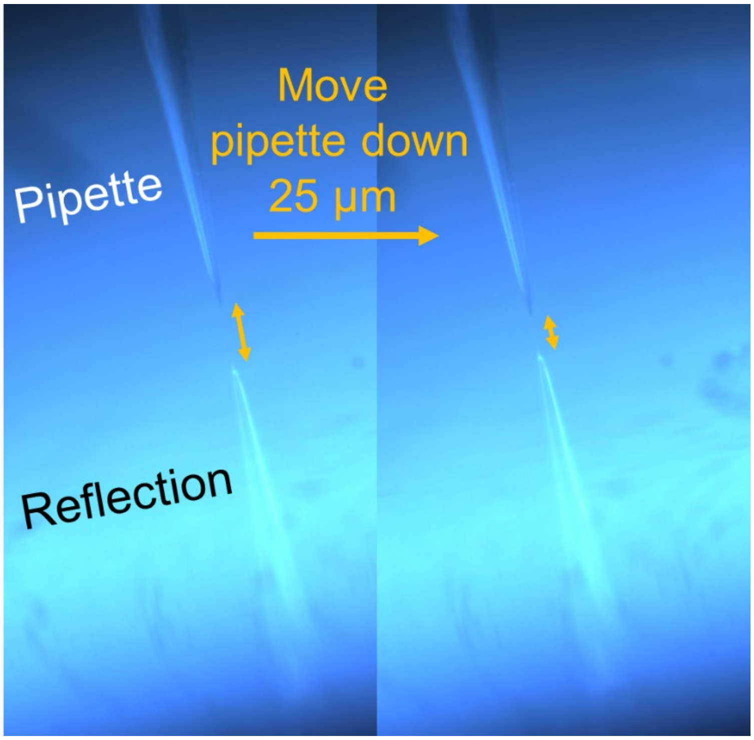

Figure S10. Side-on image of a nanopipette tip above a reflective HOPG surface before (left) and after (right) moving the pipette down 25 µm. Images were taken using a video microscope with a long working distance objective (Pixelink PL-B776U with Infinistix objective).

Figure S11 shows two images using the top-view camera of a commercial SECCM (Park Systems NX12) taken at different focal distances. In the top image has been focused on the nanopipette tip (top), which appears as a sharper point in a blurry (out of focus) background. In the bottom image the HOPG sample is in sharp focus and individual features can be observed. The *z* position of the pipette tip and sample focal planes were 15160 μm and 14966 μm, respectively, suggesting the pipette-sample separation is ~200 μm (see *Lowering the Pipette Toward Surface (Coarse Positioning)* for a discussion). Note, as it is impossible perfectly resolve the nanometric end of the pipette, such approximations should be used in a conservative manner.

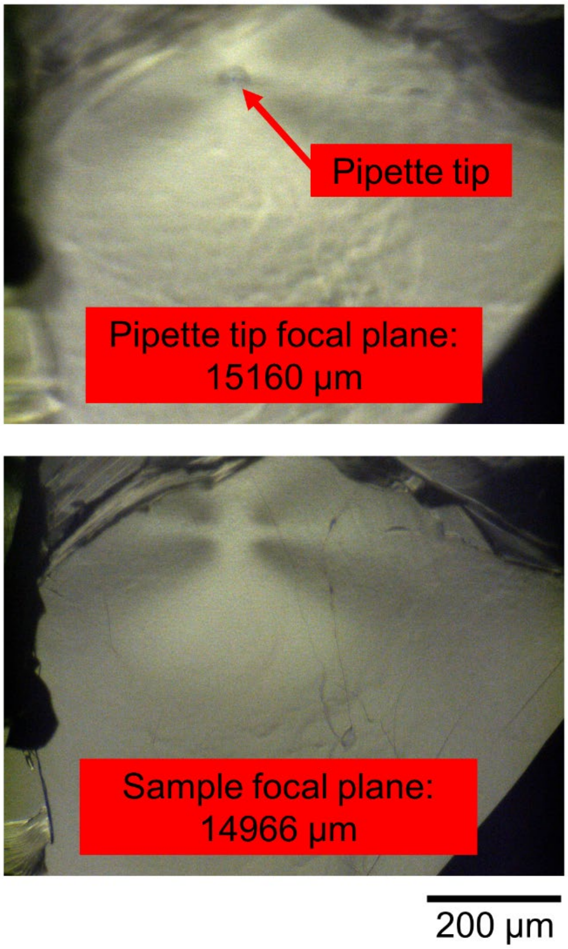

Figure S11. Top-view images of an SECCM pipette over an HOPG sample focusing on the pipette tip (top) and sample (bottom). The focal plane *z* position of the images is as labeled. Images were taken using the optics of a Park Systems NX12 microscope (UL10X ultralong working distance objective (NA 0.23, WD 50.5 mm), 5M pixel CCD camera).

S11: *z* Movement During an Inchworm Approach

Figure S12 shows the relative *z* position of the end of the nanopipette during an inchworm approach (discussed in *Running the Computer-controlled Piezo-driven Approach*). The pipette is moved toward the surface while the current is monitored. If no current trigger is observed within the piezo's z range (15 μm in this example), the piezo is fully retracted and the coarse positioning is moved down by most (e.g., 90%) of the piezo's full range. During this coarse positioning phase, the pipette is always at a greater distance from the surface than during the preceding piezo-driven approach so there is no risk of tip damage. The piezo approach-piezo retract-coarse approach cycle is then repeated until the current trigger is encountered on one of the piezo approaches (not shown here).

The inchworm approach ensures that the pipette contacts the surface in a controlled manner and allows for a greater *z* range than is afforded by the pipette range alone. Some instruments perform inchworm approaches automatically, while others require the user to control each of the individual *coarse positioning down* steps.

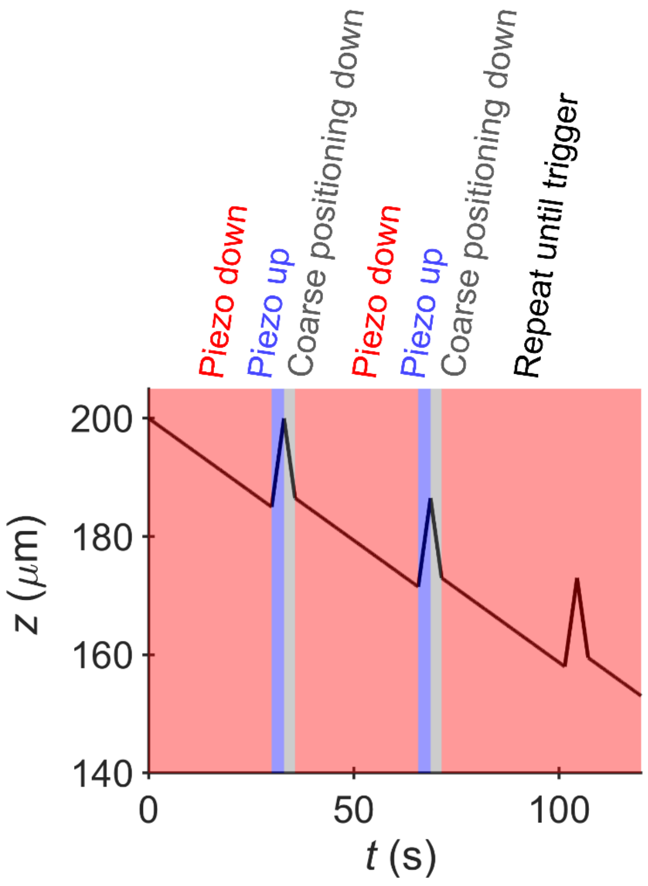

Figure S12. Schematic of the relative *z* position of the nanopipette vs time during an inchworm approach utilizing a combination of fine (piezo) and coarse positioning. The inchworm approach is discussed in *Running the Computer-controlled Piezo-driven Approach* of the main text. Piezo z range: 15 μm; coarse positioning movement: 90% full piezo range.

S12: Undesirable CV Responses

Figure S13 shows examples of undesirable voltametric responses taken at 2 different locations on an HOPG sample surface using a nanopipette (radius ≈ 500 nm) filled with 5 mM $Ru(NH_3)_6Cl_3$ with 50 mM KCl. As discussed in *Settings for Computer-Controlled Piezo-Driven Approach* of the main text, the expected current for an experiment with these parameters is ~-155 pA. The current measured at these two locations is in the nA range and much higher than the expected current, is irreproducible between cycles, and shows erratic peaks and spikes. None of these are expected from the well-behaved system under study and thus we conclude that there is tip breakage and/or droplet spreading, as discussed in *Running the Computer-Controlled Piezo-Driven Approach* of the main text. In this scenario, a new tip should be prepared for further experiments. If one is uncertain as to whether the pipette is broken, characterizing its *i-E* response in bulk solution (see *Measuring the i-t and i-E Response of a Nanopipette in Bulk Solution*) or examining the tip with optical microscopy after the experiment can be used as a diagnostic. However, when such striking deviations from the expected response are observed this practice is rarely followed.

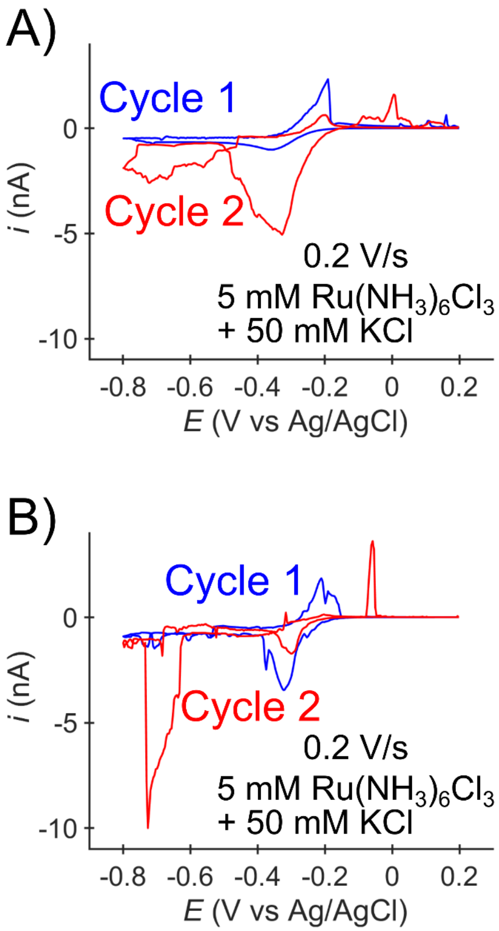

Figure S13. Undesirable cyclic voltammograms measured through the HOPG sample at two different locations (A and B) using a nanopipette (r_p ≈ 500 nm) filled with 5 mM $Ru(NH_3)_6Cl_3$ with 50 mM KCl. Two CV cycles were measured at each location, the first cycle is shown in blue and the second in red.

S13: SECCM Electrochemical Map Plotted as Isolated Circles

Figure S14 show the electrochemical map of the $Ru(NH_3)_6^{3+}$ reduction measured current at *E*=-0.8 V vs Ag/AgCl on the HOPG sample from Figure 2, where the pixels are plotted as circles representing the meniscus footprint rather than tessellated squares. As discussed in *Results of Imaging Experiments* in the main text, plotting each pixel such circles can more accurately convey where the characterization was performed. For example, in this case the non-overlapping circles indicate that only a portion of the imaging region was probed electrochemically.

In this case, we assume the meniscus footprint radius to be identical to the radius of the pipette, which is typically a fair approximation. However, if the footprint area is measured through a complementary method, e.g., post imaging of electrolyte residue through SEM[5] or AFM[6], accurate sizes may be added to each point and any variability in the droplet area will be visible.

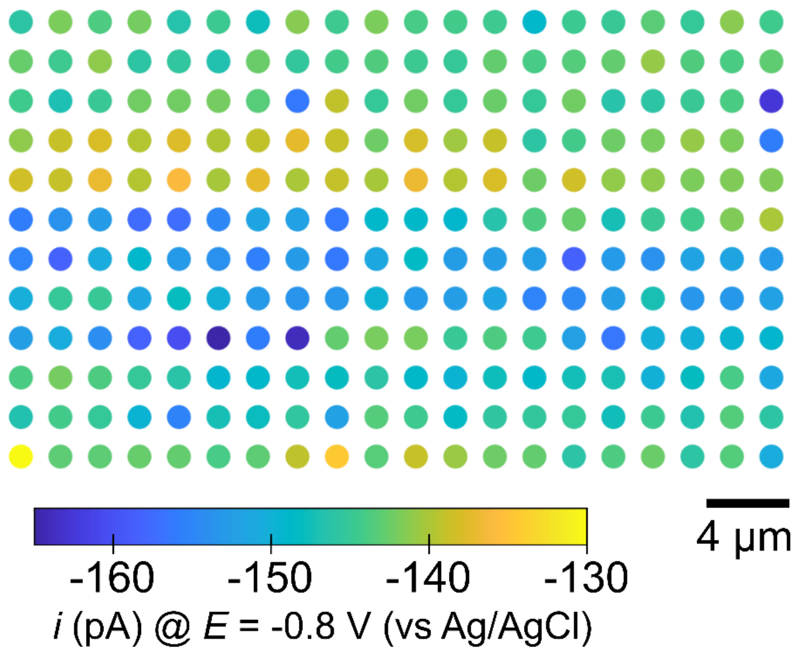

Figure S14. Data from the electrochemical image shown in Figure 2 replotted in a meniscus footprint representation. SECCM electrochemical map of the measured current, *i*, at *E* = -0.8 V vs. Ag/AgCl corresponding to the reduction of $Ru(NH_3)_6^{3+}$ on HOPG. Each pixel is been plotted as an circle representing the meniscus footprint (assumed to be equal to the pipette radius ≈ 500 nm). The solution in the pipette was 5 mM $Ru(NH_3)_6Cl_3$ with 50 mM KCl. The area imaged was 40×24 μm^2 with 20 pixels for *x* and 12 pixels for *y*. i_{thresh} = 3 pA, $E_{approach}$ = -0.8 V vs Ag/AgCl, approach speed = 0.5 µm/s, retraction distance = 2 µm, and settling time = 500 ms. See Supporting Information, section S2 for experimental details and Figure S1 for an image of the experimental setup.

S14: z-height Map from an Image Containing False-trigger Events

Figure S14 shows topographic maps obtained during the acquisition of the electrochemical image shown in Figure 16 of the main text. The two right-most pixels on the top row are from false trigger events, as confirmed by the lack of a voltammetric response (curve B, Figure 16 top). In the top version of the image, these two points appear ~4 μm higher than the rest of the surface, as would be expected by the approach terminating prematurely to a flat surface. In the lower version of the figure, only the points that represent the true height of the sample are shown and the false trigger points are neglected. In this image, a narrower *z* range is used and subsequently the topographic information (in this case a slight tilt of the otherwise flat sample) can be more clearly seen.

While isolated microns-high features are not be expected on a pristine HOPG surface or other flat samples and often are associated with false triggers, caution should be exercised to not automatically attribute such features to false triggers based on topographic images alone. Instead, comparison with the electrochemical measurement should be used to determine if the tip was in contact with the surface. In this way, genuine examples of sharp topographic features will not be overlooked.

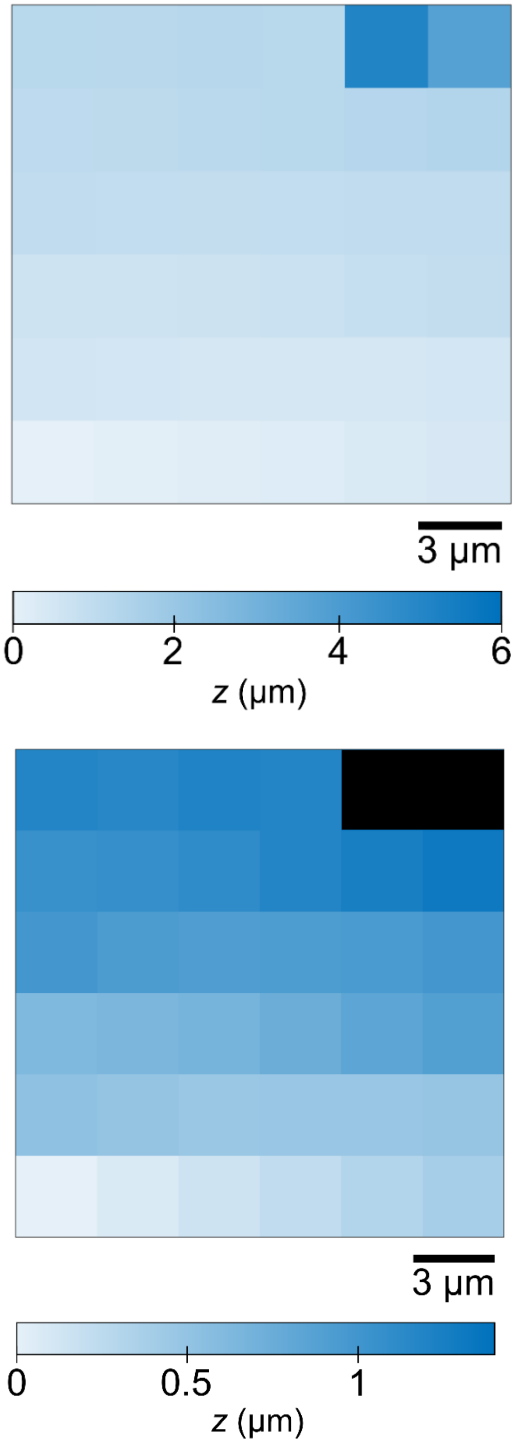

Figure S15. Z height maps corresponding to the current image presented in Figure 15 of the main text, imaging parameters as listed in Figure 15 caption. The two right-most pixels on the top row are from false trigger events. The upper image shows the height, measured as the *z* position when the current threshold was achieved during the approach. In the lower image the two false-trigger pixels are removed (black) and a narrower *z* range is used.

S15: Printable Flowcharts

Figures S16, S17, and S18 are larger, printable versions of the flowcharts shown in Figures 4, 7, and 13, respectively, of the main text.

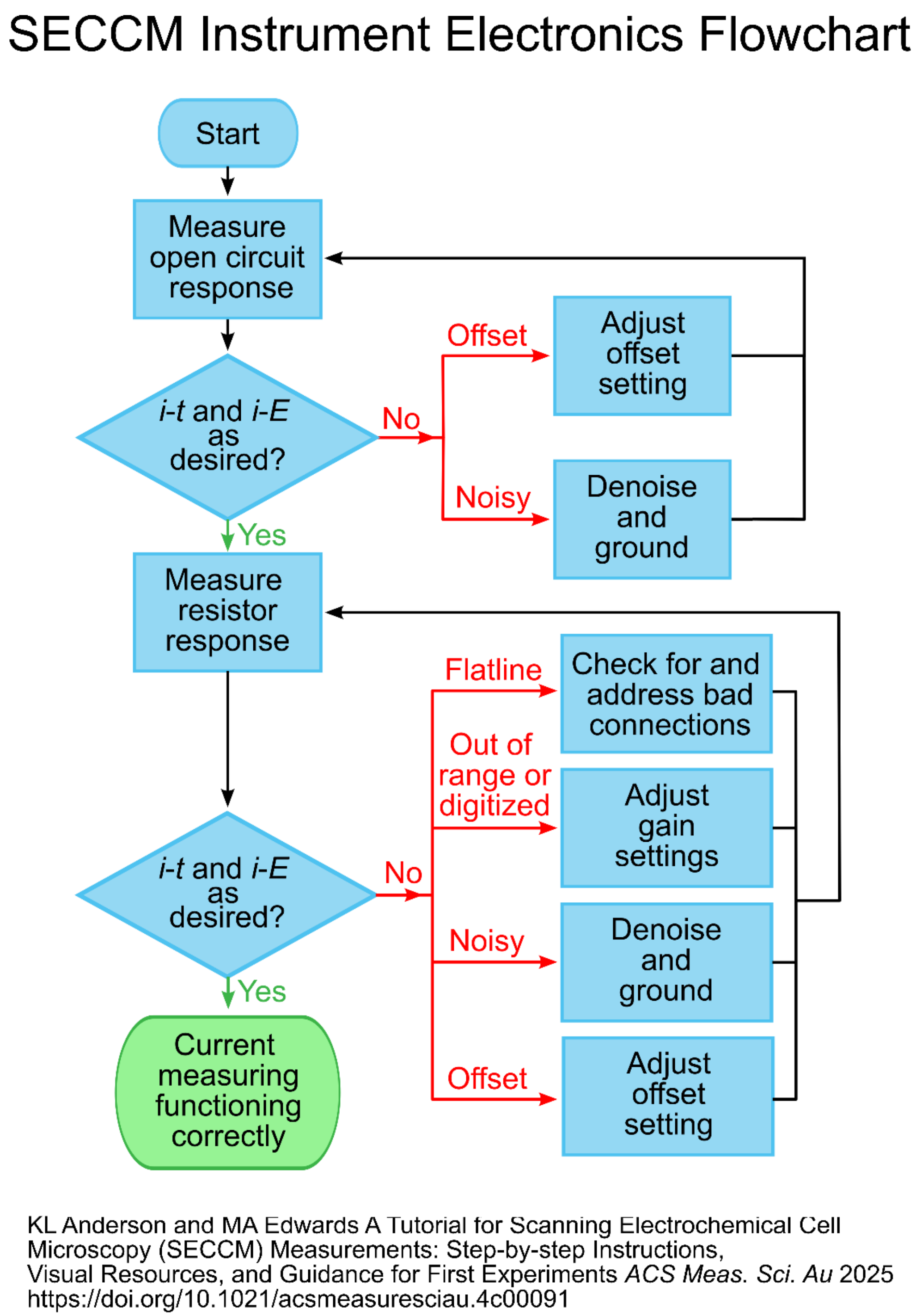

Figure S16. Printer-friendly full-page instrument electronics flowchart.

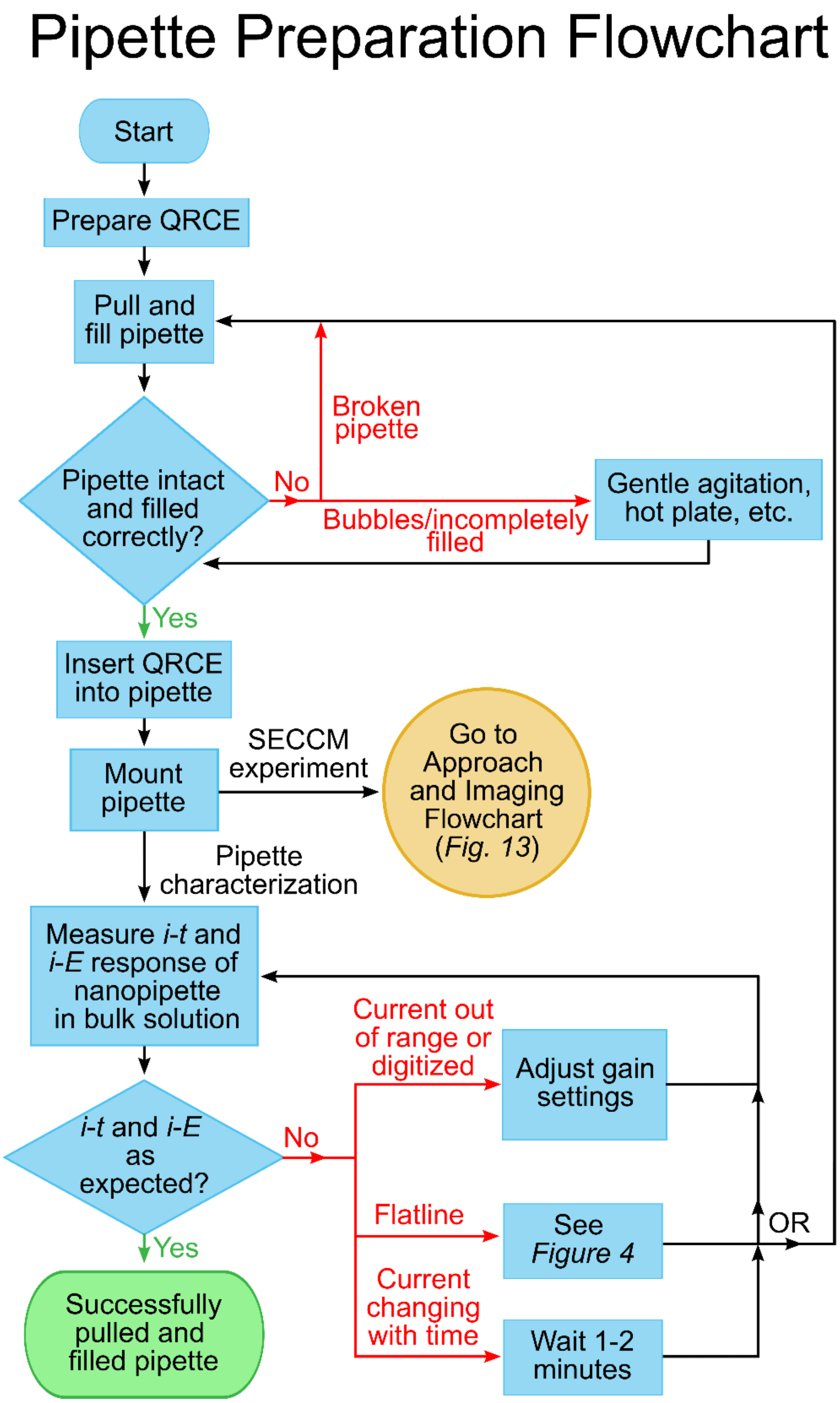

KL Anderson and MA Edwards A Tutorial for Scanning Electrochemical Cell Microscopy (SECCM) Measurements: Step-by-step Instructions, Visual Resources, and Guidance for First Experiments *ACS Meas. Sci. Au* 2025 https://doi.org/10.1021/acsmeasuresciau.4c00091

Figure S17. Printer-friendly full-page pipette preparation flowchart.

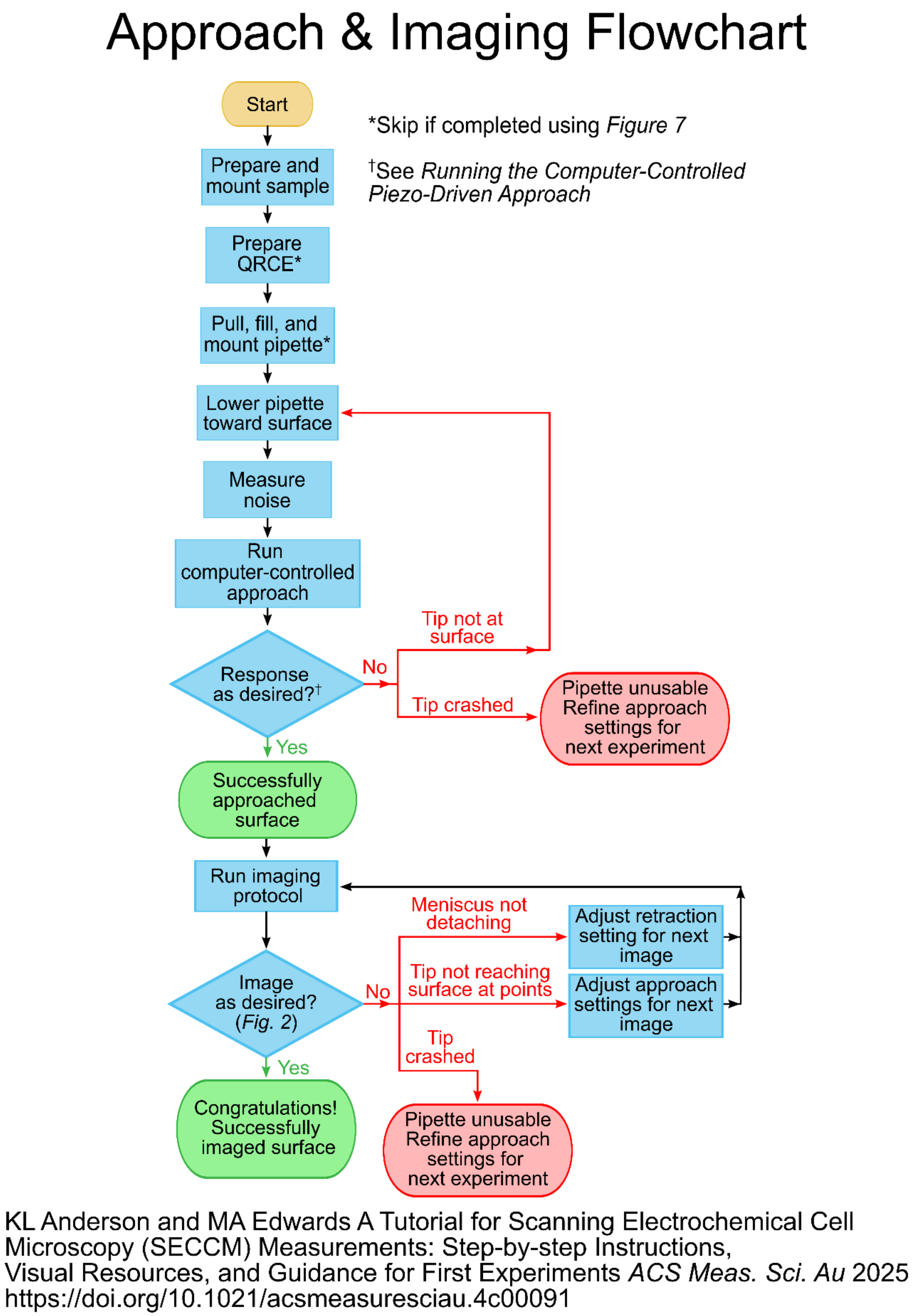

Figure S18. Printer-friendly full-page SECCM approach and imaging flowchart.

S16: Supporting Videos

Video S1: Video demonstrating the backloading method of filling a pipette described in *Filling pipettes* and shown schematically in Figure 7 of the main text. Dyed solution is used to aid visualization.

Video S2: Video demonstrating gently brushing the threads of a screw against a nanopipette to release bubbles, as discussed in *Filling pipettes*.

Video S3: Video demonstrating agitating a nanopipette with serrated tweezers to release the bubbles, as discussed in *Filling pipettes*.

S17: Vocabulary

SECCM (Scanning Electrochemical Cell Microscope): An instrument consisting of a mobile nanopipette and associated hardware used to measure the localized electrochemical response of a surface region wetted by the nanopipette meniscus. SECCM is also used to describe experiments performed with this instrument (in which case the 'M' refers to 'Microscopy').

Pipette/Nanopipette/Probe/Tip (terms used interchangeably): Glass or quartz capillary pulled to a sharp point (~20 nm to ~1 μm diameter opening), filled with electrolyte, and containing a (quasi-) reference electrode. The meniscus at the end of the tip wetting the sample forms the electrochemical cell in SECCM measurements.

Sample/Substrate/Surface/Electrode (terms used interchangeably): Material for which the local electrochemical behavior and topography are mapped by SECCM imaging.

Imaging: The process of taking SECCM measurements at an array of locations on a surface to generate maps of electrochemical behavior and topography of the sample as a function of position.

Coarse positioning: Controlling the position of the pipette relative to the surface using stepper motors, micrometers, or fine-pitch screws that have relatively low resolution (~0.5-10 μm) compared to the probe dimensions, but typically have a large range (~1-10 cm). Coarse positioners have insufficient resolution to controllably touch the meniscus at the end of the tip onto the surface but can place it near the surface (<100 μm).

(Computer-controlled piezo-driven) Approach: The process of carefully moving a SECCM pipette tip to the sample surface using a piezoelectric actuator (piezo). A computer monitors the current and ceases movement when the probe encounters the surface avoiding damage to the delicate pipette. Piezo positioning has a high resolution and limited range (<1 nm resolution and ~10-200 μm range).

False-trigger event: Triggering (terminating an approach) before the pipette has reached the surface (due to extraneous noise).

References

1 A. N. Patel, M. G. Collignon, M. A. O'Connell, W. O. Y. Hung, K. McKelvey, J. V. Macpherson and P. R. Unwin, A new view of electrochemistry at highly oriented pyrolytic graphite., *J. Am. Chem. Soc.*, 2012, **134**, 20117–30.

2 G. Jayamaha, M. Maleki, C. L. Bentley and M. Kang, Practical guidelines for the use of scanning electrochemical cell microscopy (SECCM), *Analyst*, 2024, **149**, 2542–2555.

3 I. M. Ornelas, P. R. Unwin and C. L. Bentley, High-Throughput Correlative Electrochemistry–Microscopy at a Transmission Electron Microscopy Grid Electrode, *Anal. Chem.*, 2019, **91**, 14854–14859.

4 A. J. Bard, L. R. Faulkner and H. S. White, *Electrochemical Methods: Fundamentals and Applications*, Wiley, Hoboken, NJ, 3rd edn., 2022.

5 C.-H. Chen, L. Jacobse, K. McKelvey, S. C. S. Lai, M. T. M. Koper and P. R. Unwin, Voltammetric Scanning Electrochemical Cell Microscopy: Dynamic Imaging of Hydrazine Electro-oxidation on Platinum Electrodes, *Anal. Chem.*, 2015, **87**, 5782–5789.

6 C. L. Bentley, M. Kang, F. M. Maddar, F. Li, M. Walker, J. Zhang and P. R. Unwin, Electrochemical maps and movies of the hydrogen evolution reaction on natural crystals of molybdenite (MoS_2): basal vs. edge plane activity, *Chem Sci*, 2017, **8**, 6583–6593.
